# Species diversity of edible mushrooms III: new species of *Laccaria* (*Agaricales*, *Basidiomycota*) from southwestern China based on morphology and multilocus phylogeny

**DOI:** 10.3897/mycokeys.139.175697

**Published:** 2026-08-27

**Authors:** Guo Zhao, Yi-Han Qu, Li-Yan Tang, Juan Zhang, Hao-Nan Suo, Kai-Yang Niu, Zong-Long Luo, Han-Bing Song, Song-Ming Tang

**Affiliations:** 1 College of Agriculture and Biological Science, Dali University, Dali 671003, China College of Agriculture and Biological Science, Dali University Dali China; 2 Co-Innovation Center for Cangshan Mountain and Erhai Lake Integrated Protection and Green Development of Yunnan Province, Dali University, Dali 671003, China Co-Innovation Center for Cangshan Mountain and Erhai Lake Integrated Protection and Green Development of Yunnan Province, Dali University Dali China

**Keywords:** *
Hydnangiaceae
*, phylogeny, taxonomy, two new taxa

## Abstract

Species of *Laccaria* (*Hydnangiaceae*, *Agaricales*, *Basidiomycota*) are an important group of ectomycorrhizal fungi with significant ecological and economic value. They are widely distributed. This study describes two new species of *Laccaria* based on morphological characters and molecular data. *Laccaria daliensis* is characterized by a pale orange to grayish orange pileus, grayish orange lamellae, and a stipe. *Laccaria violaceonana* is characterized by medium-sized, deep purple basidiomata and an equal stipe with an enlarged base. Phylogenetic analysis based on a combined dataset of the internal transcribed spacer ITS1–5.8S–ITS2 rDNA (nrITS), large subunit nuclear ribosomal RNA gene (nrLSU), RNA polymerase II subunit 2 (*rpb2*), and translation elongation factor 1-α (*tef1-α*) was used to provide further evidence for species delimitation. The results showed that nine *Laccaria* specimens formed four well-supported clades, representing two new species, *Laccaria daliensis* and *L. violaceonana*, and two known species, *L. acanthospora* and *L. pumila*. Detailed descriptions, illustrations, and phylogenetic analysis results of these species are provided, and the new species are compared with closely related taxa.

## Introduction

The genus *Laccaria* was established in 1883 with *L. laccata* (Scop.) Cooke as the type species. Species of this genus are typically characterized by brown to orange or purple, collybioid to omphaloid basidiomata; a convex to plane or umbilicate, distinctly hygrophanous pileus; thick, waxy, and often widely spaced lamellae; subglobose to oblong, echinulate, acyanophilous, inamyloid basidiospores; and the presence of clamp connections (Berkeley and Broome 1883; Singer 1986; Mueller 1991, 1992; Wang et al. 2004; Luo et al. 2016; Popa et al. 2016; Latha et al. 2019; Razzaq et al. 2023; Tang et al. 2024, 2025; Zhang et al. 2024). To date, more than 100 species of *Laccaria* have been recorded worldwide (Kirk et al. 2008; He et al. 2019; Corrales et al. 2020; Li 2020; Zhang et al. 2023; Tang et al. 2024). The early taxonomy of the genus, however, was predominantly developed from studies in Europe and North America (Singer 1967; Lahaie 1981; Pázmány 1994; Montoya et al. 2015; Wilson et al. 2015, 2017).

In China, early research on *Laccaria* primarily relied on morphological characteristics. With the development and application of molecular systematics, particularly since the comprehensive study by Wang et al. (2004), the species diversity of this genus within the country has gradually become clearer. To date, 45 *Laccaria* species have been recorded nationwide across 27 provinces and autonomous regions. Yunnan and Xizang have been identified as biodiversity hotspots, with 22 and 11 species reported, respectively (Wang et al. 2004; Wilson et al. 2013; Luo et al. 2016; Mu and Bau 2016; Vincenot et al. 2017; Li 2020; Cui et al. 2021; Wang et al. 2022; Zhang et al. 2023; Tang et al. 2024, 2025; Zhang et al. 2024). This genus is a typical ectomycorrhizal group, and all currently known species are considered edible (Zhang et al. 2023; Tang et al. 2025). For instance, the four species investigated in the present study are also edible. According to the “Checklist of Chinese Ectomycorrhizal Edible Mushrooms”, species such as *L. acanthospora* A.W. Wilson & G.M. Muell. and *L. angustilamella* Zhu L. Yang & L. Wang are edible (Wei et al. 2021). Among the 11 *Laccaria* species included in the “Checklist of Edible Fungi in China”, four have been confirmed to possess antitumor activity (Dai et al. 2008, 2010). In Asia, certain larger species, such as *L. yunnanensis* Popa, Rexer, Donges, Zhu L. Yang & G. Kost and *L. moshuijun* Popa & Zhu L. Yang, are highly valued for their excellent culinary qualities (Tang et al. 2025).

However, species identification within *Laccaria* has long been challenging. The morphology of the basidiomata, especially key taxonomic characters such as color, exhibits high plasticity and is significantly influenced by environmental humidity. Under moist conditions, different species can display their distinct and diagnostic colors, whereas under dry conditions, the basidiomata generally dehydrate and turn grayish brown to earth brown, making species difficult to distinguish (Wilson et al. 2017; Cui et al. 2021; Hu et al. 2022; Wang et al. 2022). Consequently, while a few species can be identified by distinctive morphological features, accurate delimitation of most species necessitates reliance on molecular systematic evidence.

With the advancement of phylogenetic studies utilizing multiple gene fragments, such as the nuclear ribosomal internal transcribed spacer (nrITS) region, large subunit of nuclear ribosomal RNA gene (nrLSU), RNA polymerase II subunit 2 (*rpb2*), and translation elongation factor 1-α (*tef1-α*), substantial sequence data have accumulated in public databases (Sheedy et al. 2013; Wilson et al. 2013, 2017; Popa et al. 2014, 2016; Cho et al. 2020). This has enabled the accurate identification of *Laccaria* species using molecular data. Molecular systematic methods have thus become an indispensable tool for resolving taxonomic difficulties within the genus and for discovering and recognizing new species (Bruns et al. 1991; Mueller and Ammirati 1993; Gardes and Bruns 1993; Taylor et al. 2000; Osmundson et al. 2005; Popa et al. 2016; Tang et al. 2024, 2025).

In this study, based on integrated morphological and multilocus phylogenetic analyses of specimens collected from northwestern Yunnan Province, a taxonomic investigation of the genus *Laccaria* was conducted. Two new species are described and two known species are reported, with the aim of enhancing understanding of *Laccaria* diversity in this region and contributing new data to the taxonomy and phylogeny of the genus.

## Material and methods

### Morphological study

A total of nine specimens used in this study were collected from southwestern China. During field surveys, *in situ* photographs of fresh basidiomata were taken to document macromorphological features, and detailed field data, including collection localities, dates, habitats, elevations, and geographic coordinates (latitude and longitude), were recorded concurrently (Rathnayaka et al. 2025). Color codes were assigned following the Color Hexa website (http://www.colorhexa.com), and morphological descriptions followed Vellinga (Bas et al. 1988). Specimens were thoroughly dried at 50 °C in a food dehydrator (Hu et al. 2022), subsequently stored in sealed plastic bags, and deposited in the Herbarium of Cryptogams, Kunming Institute of Botany, Chinese Academy of Sciences (**KUN-HKAS**), as well as at Dali University. For microscopic examination, dried material was sectioned under a stereomicroscope and transferred to glass slides. The sections were then treated with a 5% potassium hydroxide (KOH) solution and stained with 1% Congo red prior to observation. Microscopic features, including basidia, basidiospores, and cystidia, were observed and photographed using a Nikon Eclipse 80i compound microscope at magnifications up to ×1000. For low-vacuum scanning electron microscopy (SEM), fragments of dried lamellae bearing basidiospores were mounted on aluminum stubs using double-sided conductive tape, sputter-coated with gold, and examined at ×10,000 magnification with a Hitachi TM4000 PlusII microscope (Hitachi, Japan) at the Dali University Analysis and Testing Center. Basidiospore dimensions and surface ornamentation (echinulae) were measured from SEM images with an accuracy of 0.1 µm. The notation [*x*/*y*/*z*] indicates that measurements are based on *x* basidiospores from *y* basidiomata belonging to *z* collections. At least 50 basidiospores, 20 basidia, and 20 cystidia (where present) were measured per basidioma. Basidiospore dimensions, excluding ornamentation, are presented as (*a*–) *b*–*c* (–*d*) µm, where ‘*a*’ and ‘*d*’ represent the absolute minimum and maximum of all measurements, respectively, and ‘*b–c*’ denotes the range encompassing 95% of the measured values. The mean value is indicated by “av.” placed between the size limits. The length-to-width ratio of an individual basidiospore is given as *Q*; the mean *Q* value for all basidiospores measured (± sample standard deviation) is denoted as *Q*m. Measurements of other microscopic structures are presented as the minimum-to-maximum length or width (excluding outliers), with the mean value (“av.”) provided between the extremes.

### DNA extraction, PCR amplification, and sequencing

Genomic DNA was extracted from dried specimens using the Ezup Column Fungi Genomic DNA Extraction Kit (Sangon Biotech, China) following the manufacturer’s protocol. The PCR primer pairs were as follows: nrITS, ITS1/ITS4 (White et al. 1990); nrLSU, LR5/LR0R (Vilgalys and Hester 1990); *rpb2*, *rpb2*-5F/*rpb2*-7cR (Liu et al. 1999); and *tef1-α*, *tef1*-983F/*tef1*-2218R (Rehner and Buckley 2005). Amplification of nrITS, nrLSU, *rpb2*, and *tef1-α* was performed in 25 µL reaction systems containing 12.5 µL 2× Taq Plus Master Mix II (Vazyme Biotech Co., Ltd., China), 9.5 μL ddH_2_O, 1 µL each of the forward and reverse primers, and 1 µL DNA. The amplifications were conducted on a C1000 Thermal Cycler (Bio-Rad, Beijing, China) using the following programs: for nrITS and nrLSU, initial denaturation at 94 °C for 5 min; 35 cycles of denaturation at 94 °C for 30 s, annealing at 48 °C for 30 s, and extension at 72 °C for 90 s; followed by a final extension at 72 °C for 10 min (White et al. 1990). For *rpb2* and *tef1-α*, the program consisted of initial denaturation at 95 °C for 5 min; 35 cycles of denaturation at 95 °C for 60 s, annealing at 50 °C for 90 s, and extension at 72 °C for 60 s; followed by a final extension at 72 °C for 10 min. Successfully amplified PCR products were sent to Sangon Biotech (Shanghai, China) for sequencing.

### Sequence alignment and phylogenetic analyses

The newly determined nucleotide sequences of *Laccaria* species have been deposited in the NCBI database (https://submit.ncbi.nlm.nih.gov/). To verify sequence identity and screen for potential contamination, all sequences were analyzed using the Basic Local Alignment Search Tool (BLAST v. 2.17.0) (NCBI, https://blast.ncbi.nlm.nih.gov), confirming their taxonomic placement within the genus *Laccaria*. Representative sequences were then selected from recent literature to assemble clusters of closely related homologs.

For sequence editing and alignment processing, raw sequence chromatograms were inspected and manually edited using BioEdit v. 7 (Hall et al. 2011a) to correct ambiguous base calls and trim low-quality regions at both ends. Cleaned sequences were aligned using MAFFT v. 7 (Katoh and Standley 2013) with the L-INS-i strategy, which is recommended for sequences with conserved motifs. After alignment, manual refinement was performed in BioEdit v. 7 (Hall et al. 2011a) to optimize positional homology, and ambiguously aligned regions were excluded to avoid introducing erroneous phylogenetic signals. The final alignments of nrITS, nrLSU, *rpb2*, and *tef1-α* comprised 617 bp, 693 bp, 1,124 bp, and 1,136 bp, respectively.

Maximum likelihood (ML) phylogenetic analysis was conducted using RAxML-HPC2 v. 8.2.3 (Stamatakis 2014) on the CIPRES Science Gateway v. 3.3 (Miller et al. 2010) platform under the GTR+G+I model of evolution, with 1,000 rapid bootstrap replicates per gene partition. Given that no significant topological incongruences were detected among the individual gene trees (supported by pairwise Shimodaira–Hasegawa [SH] tests, *p* > 0.05), the four single-gene alignments were concatenated into a single dataset using Sequence Matrix-Windows-1.7.8 (Vaidya et al. 2011).

Bayesian phylogenetic analysis was performed with MrBayes v. 3.2 (Ronquist et al. 2011). The best-fit nucleotide substitution model for each partition was selected using MrModeltest v.2.3 (Nylander 2004) based on the Akaike information criterion (AIC). The selected models were GTR+I+G for nrITS, nrLSU, and *tef1-α* [Lset nst = 6, rates = invgamma; Prset statefreqpr = dirichlet (1, 1, 1, 1)] and HKY+I+G for *rpb2* [Lset nst = 2, rates = invgamma; Prset statefreqpr = dirichlet (1, 1, 1, 1)]. Two independent runs of six Markov chain Monte Carlo (MCMC) chains each were conducted for 10,000,000 generations, with trees sampled every 100 generations. Convergence was assessed by ensuring that the average standard deviation of split frequencies fell below 0.01 and by verifying that the potential scale reduction factor (PSRF) approached 1.0 for all parameters. The first 25% of trees were discarded as burn-in (Rannala and Yang 1996). The runs were terminated automatically using the stoprule command once the average standard deviation of split frequencies fell below 0.01.

A clade was considered strongly supported if it received bootstrap support (BS) ≥ 75% in the ML analysis and a Bayesian posterior probability (PP) ≥ 0.90. The generic names of *Laccaria* and *Mythicomyces* used in this study are abbreviated as “*L.*” and “*M.*,” respectively. The alignment was submitted to Figshare (https://doi.org/10.6084/m9.figshare.30413050).

## Results

### Phylogenetic analyses

The combined nrITS, nrLSU, *rpb2*, and *tef1-α* sequence dataset comprised 166 specimens (Table 1) and 3,570 characters, including gaps (nrITS: 1–617 bp; nrLSU: 618–1,310 bp; *rpb2*: 1,311–2,434 bp; and *tef1-α*: 2,435–3,570 bp). Based on previous phylogenetic studies (Wilson et al. 2013, 2017; Popa et al. 2014, 2016; Luo et al. 2016; Cho et al. 2018; Li 2020; Cui et al. 2021; Zhang et al. 2023), *Mythicomyces corneipes* (Fr.) Redhead & A.H. Sm. was selected as the outgroup. RAxML and Bayesian analyses yielded topologies that were generally congruent. The best RAxML tree, with a final likelihood value of −23,210.818382, is presented. The matrix contained 1,679 distinct alignment patterns, with 54.02% of characters undetermined or missing. Estimated base frequencies were as follows: A = 0.255912, C = 0.218322, G = 0.246813, and T = 0.278953; substitution rates were AC = 1.234966, AG = 5.341752, AT = 1.452789, CG = 0.938279, CT = 7.635155, and GT = 1.000000; and the gamma distribution shape parameter was *α* = 0.273343.

**Table 1.** Names, specimen numbers, and GenBank accession numbers for the nrITS, nrLSU, *rpb2*, and *tef1-α* sequences of the *Laccaria* taxa used in this study, along with relevant references. Species newly described in this study are indicated in bold, and an asterisk (*) following the specimen number denotes the holotype specimen.

**Species name**	**Sample no**.	**GenBank accession**				**References**
		**ITS**	**LSU**	***rpb2***	***tef1-α***	
*Laccaria acanthospora*	HKAS45998	KU685719	KU685870	KU686069	-	Wilson et al. 2013
	AWW485*	JX504102	JX504186	-	-	Wilson et al. 2013
	**HKAS147006**	**PV927340**	**PV857763**	**PX943789**	**-**	**This study**
*L. affinis*	GDOR5565	PQ642690	-	PQ653981	-	Dovana et al. 2025
	GDOR5564	PQ642691	-	-	-	Dovana et al. 2025
*L. alba*	AWW438	JX504094	JX504178	KU685912	KU686072	Wilson et al. 2013
	TPML20120807-69	MG519542	MG519583	MG551616	MG551649	Cho et al. 2018
*L. albifolia*	GDOR5569*	PQ642680	PQ642694	-	PQ653979	Dovana et al. 2025
	GDOR 5573	PQ642683	PQ642696	PQ653980	PQ653978	Dovana et al. 2025
*L. albostipitata*	HKAS 123300 *	PQ651565	PQ753313	PQ753328	PQ753342	Tang et al. 2025
	HKAS 123285	PQ651566	PQ753314	PQ753329	PQ753343	Tang et al. 2025
*L. ambigua*	PDD89696*	KU685725	KU685876	KU686018	KU686132	Wilson et al. 2017
*L. amethysteo-occidentalis*	AWW556	JX504107	JX504191	KU685919	-	Wilson et al. 2013
	KG P40*	DQ822817	-	-	-	Peay et al. 2007
*L. amethystina*	GMM7633	JX504154	JX504228	-	-	Wilson et al. 2017
	GMM7621	JX504150	JX504224	KU686046	KU686152	Wilson et al. 2013
*L. angustilamella*	BAP226	JX504118	JX504201	-	-	Wilson et al. 2013
*L. anthracina*	HMAS254678*	KX496973	-	-	-	Direct Submission
	HMAS260494	KX496974	-	-	-	Direct Submission
*L. araneosa*	SFC2013091721*	MG519549	MG519589	MG551622	MG551655	Cho et al. 2018
	TPML20120912-40*	MG519548	MG519588	MG551621	MG551654	Cho et al. 2018
*L. aurantia*	KUNF78557*	NR154113	-	-	-	Popa et al. 2014
	MBFB001109	JQ681209	-	-	-	Popa et al. 2014
*L. aurantiaca*	HKAS 123244 *	PQ651572	PQ720997	PQ753335	PQ753349	Tang et al. 2025
	HKAS 123246	PQ651573	PQ720998	PQ753336	PQ753350	Tang et al. 2025
*L. bicolor*	HKAS44062	JX504159	JX504235	KU686068	-	Wilson et al. 2013
	KA130253	MG519524	MG519570	MG551599	MG551636	Cho et al. 2018
*L. bullipellis*	AWW465*	JX504100	JX504184	KU685914	-	Wilson et al. 2013
*L. brunnea*	HKAS 123286 *	PQ651574	PQ721003	PQ753337	PQ753351	Tang et al. 2025
	HKAS 144550	PQ651578	PQ721007	PQ753341	PQ753355	Tang et al. 2025
*L. canaliculata*	GMM7267	JX504137	JX504213	KU685960	KU686093	Wilson et al. 2013
	GMM7251	KU685669	KU685812	KU686090	KU685955	Wilson et al. 2013
*L. carminostipes*	MHHNU 11944	PV300443	PV300522	PV467480	PV339966	Xu et al. 2025
	MHHNU 31553*	PV300441	-	PV467478	PV339964	Xu et al. 2025
*L. cinnabarina*	KUN-HKAS79704	OR722589	OR722600	PP171547	PP171557	Li et al. 2024
	KUN-HKAS80885*	OR722587	OR722595	-	-	Li et al. 2024
***L. daliensis***	HKAS147004*	**PV850075**	**PV857700**	**PX425769**	**-**	**This study**
	**HKAS147005**	**PV856217**	**PV857614**	**PX943787**	**PX607686**	**This study**
	**HKAS 147007**	**PX380966**	**PV857675**	**PX943788**	**PX607685**	**This study**
*L. dallingii*	Corrales 543	MT279238	MT279213	MT431187	MT436076	Corrales et al. 2020
	Corrales 571*	MT279240	MT279214	-	-	Corrales et al. 2020
*L. darjeelingensis*	CUHAM788*	OQ607624	-	-	-	Thapa et al. 2024
*L. fagacicola*	HKAS90435*	MW540806	-	-	-	Cui et al. 2021
	HKAS107731	MW540807	-	-	-	Cui et al. 2021
*L. fengkaiensis*	HKAS106739*	MN585657	MN621238	-	-	Li 2020
	HKAS106741	MN585658	-	-	-	Li 2020
*L. fortunensis*	Corrales 74*	MT279246	-	-	-	Corrales et al. 2020
	Corrales 75	MT279247	-	-	-	Corrales et al. 2020
*L. fulvogrisea*	KUN-F78556*	NR154114	-	-	-	Popa et al. 2014
	KUN-FB-101105	JQ681210	-	-	-	Popa et al. 2014
*L. gomezii*	F1102433	-	MT279205	MT431180	-	Corrales et al. 2020
	GMM7173	MT279227	MT279207	MT431182	MT436071	Corrales et al. 2020
*L. griseolilacina*	SFC20190919-48*	MT322981	MT322983	MT333269	MT333266	Osmundson et al. 2005
*L. guizhouensis*	HMAS352265*	OP244890	-	-	-	Zhang et al. 2024
	HMAS352266	OP244891	-	-	-	Zhang et al. 2024
*L. galerinoides*	F1081213	KU685634	KU685778	KU686078	KU685929	Wilson et al. 2017
	F1080983	KU685632	KU685776	KU686077	KU685927	Wilson et al. 2017
*L. glabripes*	GMM7521	KU685708	KU685849	KU685991	KU686117	Wilson et al. 2017
	GMM7534	KU685711	KU685852	-	-	Wilson et al. 2017
*L. himalayensis*	AWW463	JX504098	JX504182	KU685913	-	Wilson et al. 2013
	AWW484*	JX504101	JX504185	KU685915	-	Wilson et al. 2013
*L. indohimalayana*	KD 17-46	MK575505	-	-	-	Wang et al. 2019
	KD 17-20	MK584157	-	-	-	Wang et al. 2019
*L. infundibuliformis*	CUHAM786	OQ607560	-	-	-	Thapa et al. 2024
*L. japonica*	F64167*	KU962988	-	-	-	Vincenot et al. 2017
	SFC2012072212	MG519518	MG519566	MG551595	MG551633	Cho et al. 2018
*L. longipes*	F1092175	KU685637	KU685780	-	-	Wilson et al. 2017
	MQ18R253-QFB30769	MN992191	-	-	-	Direct Submission
*L. laccata*	C4083, BAFC (epitype)	PV700553	PV700591	PV835003	-	Dovana et al. 2025
	GDOR5794	PV700554	-	PV835008	-	Dovana et al. 2025
	GDOR5788	PV700555	PV700592	PV835004	-	Dovana et al. 2025
*L. lateritia*	RGB 166658	JN235950	-	-	-	Direct Submission
	RGB 166659	JN235949	-	-	-	Direct Submission
*L. longistriata*	KUN-HKAS123801*	OQ396730	-	OR347685	-	Li et al. 2024
	KUN-HKAS115989	OQ396729	-	OR347682	OR347688	Li et al. 2024
*L. macrocystidiata*	GDOR5075 (epitype)	MW584890	-	-	-	Dovana et al. 2021
*L. mangshanensis*	MHHNU 8856	PV300438	PV300519	PV467476	PV339962	Xu et al. 2025
	MHHNU 8850*	PV300437	PV300518	PV467475	PV339961	Xu et al. 2025
*L. miniata*	GDGM76043*	OR689440	OR785476	-	-	Zhang et al. 2024
*L. montana*	C5442*	OR419936	-	-	-	Direct Submission
	M5464	OR419935	-	-	-	Direct Submission
*L. moshuijun*	HKAS 93732*	KU962989	-	-	-	Vincenot et al. 2017
	MB-001113	KU962985	-	-	-	Vincenot et al. 2017
*L. murina*	ASIS216	MG519553	-	-	-	Cho et al. 2018
	ASIS24249	MG519552	MG519592	MG551625	MG551658	Cho et al. 2018
*L. nanlingensis*	GDGM 84954*	OR689442	OR785478	OR835199	OR826273	Zhang et al. 2023
	GDGM 84949	OR689441	OR785477	OR835198	OR826274	Zhang et al. 2023
*L. negrimarginata*	GMM7631	JX504152	JX504226	-	-	Wilson et al. 2013
*L. neovinaceoavellanea*	GDGM52852*	OR689447	OR785479	-	-	Zhang et al. 2023
	GDGM53063	OR689448	OR785480	-	-	Zhang et al. 2023
*L. nitrophila*	Corrales 467	MT279233	-	-	-	Corrales et al. 2020
	Corrales 595*	MT279236	MT279211	MT431186	MT436074	Corrales et al. 2020
*L. nobilis*	F1091206	KU685636	KU685779	-	-	Wilson et al. 2017
	AWW584	JX504110	JX504193	KU685922	-	Wilson et al. 2013
*L. ohiensis*	KH_07192006_1	KU685720	KU685871	KU686014	-	Wilson et al. 2017
	GMM7028	KU685653	KU685796	KU685939	-	Wilson et al. 2017
*L. oblongospora*	ObiFr	GQ406466	-	-	-	Vincenot et al. 2017
*L. ochropurpurea*	PRL4777	KU685733	KU685883	KU686025	-	Wilson et al. 2017
*L. pallidorosea*	KUN-HKAS53170	MW540809	-	-	-	Cui et al. 2021
	KUN-HKAS107730*	MW540808	-	-	-	Cui et al. 2021
*L. parva*	SFC20120919-05*	MG519529	MG519573	MG551604	MG551640	Cho et al. 2018
	SFC20120906-01	MG519527	MG519572	MG551639	MG551602	Cho et al. 2018
*L. pakistanica*	LAH38689 *	PV577726	PV600478	-	-	Rafique et al. 2025
	LAH38690	PV577725	-	-	-	Rafique et al. 2025
*L. populina*	GDOR411*	MN871894	MN873018	-	-	Boonmee et al. 2021
	GDOR 408	MN871895	MN873017	-	-	Boonmee et al. 2021
*L. prava*	HKAS106742*	MN585660	-	-	-	Li 2020
	HKAS106745	MN585661	-	-	-	Li 2020
*L. proxima*	F1081079	KU685633	KU685777	KU685928	-	Wilson et al. 2017
	GMM7584	KU685717	KU685858	KU685999	KU686120	Wilson et al. 2017
*L. pseudoalba*	MFLU 22-0106*	ON557377	ON556492	ON598886	-	Tang et al. 2024
	HKAS 110664	ON557376	ON556491	ON598887	ON598894	Tang et al. 2024
*L. pseudomontana*	Cripps 1771	DQ149870	-	-	-	Osmundson et al. 2005
	pse1625*	DQ149871	-	-	-	Osmundson et al. 2005
*L. pumila*	pum1252	DQ149864	-	-	-	Osmundson et al. 2005
L. aff. montana	AWW446	JX504097	JX504181	KU686054	KU686157	Wilson et al. 2013
*L. pumila*	GMM7637	KM067861	JX504229	-	KU686158	Wilson et al. 2013
	HMAS293222	ON877220	-	-	-	Wang et al. 2022
	**HKAS147008**	**PV927342**	**PV866769**	**PX943783**	**-**	**This study**
	**HKAS147009**	**PV927341**	**PV866770**	**PX943784**	**-**	**This study**
*L. roseoalbescens*	LM5099*	KJ874328	KJ874331	-	-	Montoya et al. 2015
	VB4678	KJ590509	KJ590510	-	-	Montoya et al. 2015
*L. rubra*	HKAS 123291 *	PQ651570	PQ776317	PQ753333	PQ753347	Tang et al. 2025
	HKAS 123292	PQ651571	PQ776318	PQ753334	PQ753348	Tang et al. 2025
*L. rubroalba*	KUN-HKA 90753*	KX449358	-	-	-	Luo et al. 2016
	KUN-HKA 90758	KX449357	-	-	-	Luo et al. 2016
*L. rufobrunnea*	GDGM82878*	OR689443	OR785482	OR835197	OR826272	Zhang et al. 2023
	GDGM89627	OR689444	OR785483	-	-	Zhang et al. 2023
*L. salmonicolor*	GMM7596*	JX504143	JX504218	KU686045	KU686151	Wilson et al. 2013
	GMM7602	JX504145	-	-	-	Wilson et al. 2013
*L. sinolateritia*	MHHNU 11958	PV300445	PV300524	PV467482	PV339968	Xu et al. 2025
	MHHNU 11956*	PV300444	PV300523	PV467481	PV339967	Xu et al. 2025
*L. spinulosa*	KUN-HKAS129615*	OR722591	OR722598	PP171551	-	Li et al. 2024
	KUN-HKAS90444	OR722590	OR722597	PP171553	-	Li et al. 2024
*L. stellata*	Corrales27	MT279231	MT279210	-	MT431185	Corrales et al. 2020
	SYC 207	KP877339	-	-	-	Popa et al. 2016
*L. striatula*	1475	OQ612526	-	-	-	Cortese and Horton 2024
	CNV105	MT345281	-	-	-	Direct Submission
*L. squarrosa*	DM63*	MF669958	MF669965	-	-	Ramos et al. 2017
	DM121	MF669960	MF669967	-	-	Ramos et al. 2017
*L. subroseoalbescens*	MFLU23-0339*	PP785397	PP789598	-	-	Tang et al. 2024
	MFLU23-0340	PP785398	PP789599	-	-	Tang et al. 2024
*L. torosa*	SFC2015090217*	MG519561	MG519598	MG551631	MG551664	Cho et al. 2018
	KA12-1306	MG519562	-	-	-	Cho et al. 2018
*L. tortilis*	ASIS22273*	MG519533	MG519576	MG551608	MG551644	Cho et al. 2018
	GMM7635	JX504155	KU685906	KU686053	KU686156	Wilson et al. 2013
*L. trichodermophora*	F1111951	KU685640	KU685784	KU686063	-	Wilson et al. 2017
	GMM7733	JX504157	-	KU686013	-	Wilson et al. 2013
	TENN42523*	DQ149868	-	-	-	Osmundson et al. 2005
*L. trullisata*	PRL7587	JX504170	JX504247	KU686047	KU686153	Wilson et al. 2013
	WCG2075	KM067894	-	-	-	Wilson et al. 2015
*L. umbilicata*	GDGM82883	OR689445	OR785485	OR835194	OR826270	Zhang et al. 2023
	GDGM82911*	OR689446	OR785486	OR835192	OR826268	Zhang et al. 2023
*L. versiforma*	KNU2012100803	MG519560	MG519597	MG551630	MG551663	Cho et al. 2018
	SFC20120926-01*	MG519556	MG519594	MG551627	MG551660	Cho et al. 2018
***L. violaceonana***	**HKAS123240**	**PV857589**	**PV857717**	**PX943786**	**-**	**This study**
	HKAS123243*	**PV857581**	**PV857703**	-	**-**	**This study**
	**HKAS 123290**	**PV857576**	**PV857701**	**PX943785**	**-**	**This study**
*L. vinaceoavellanea*	A2986	JN942810	JN939738	JN993520	-	Direct Submission
	SFC20150810-10	MG519539	MG519580	MG551614	MG551646	Cho et al. 2018
*L. violaceonigra*	GMM7520	KU685707	KU685848	KU685990	-	Wilson et al. 2017
*L. violaceotincta*	CAL1389*	MK141034	-	-	-	Latha et al. 2019
*L. yunnanensis*	KUNF78558*	NR154115	-	-	-	Popa et al. 2014
	MFLU 22-0107	ON557374	ON556488	-	ON598892	Tang et al. 2024
*Mythicomyces corneipes*	ES11.10.2. A	KC964108	-	-	-	Popa et al. 2016
	AFTOLID972	DQ404393	AY745707	DQ408110	DQ029197	Popa et al. 2016

Note: The “-” denotes the unavailability of the sequences.

**Figure 1.**
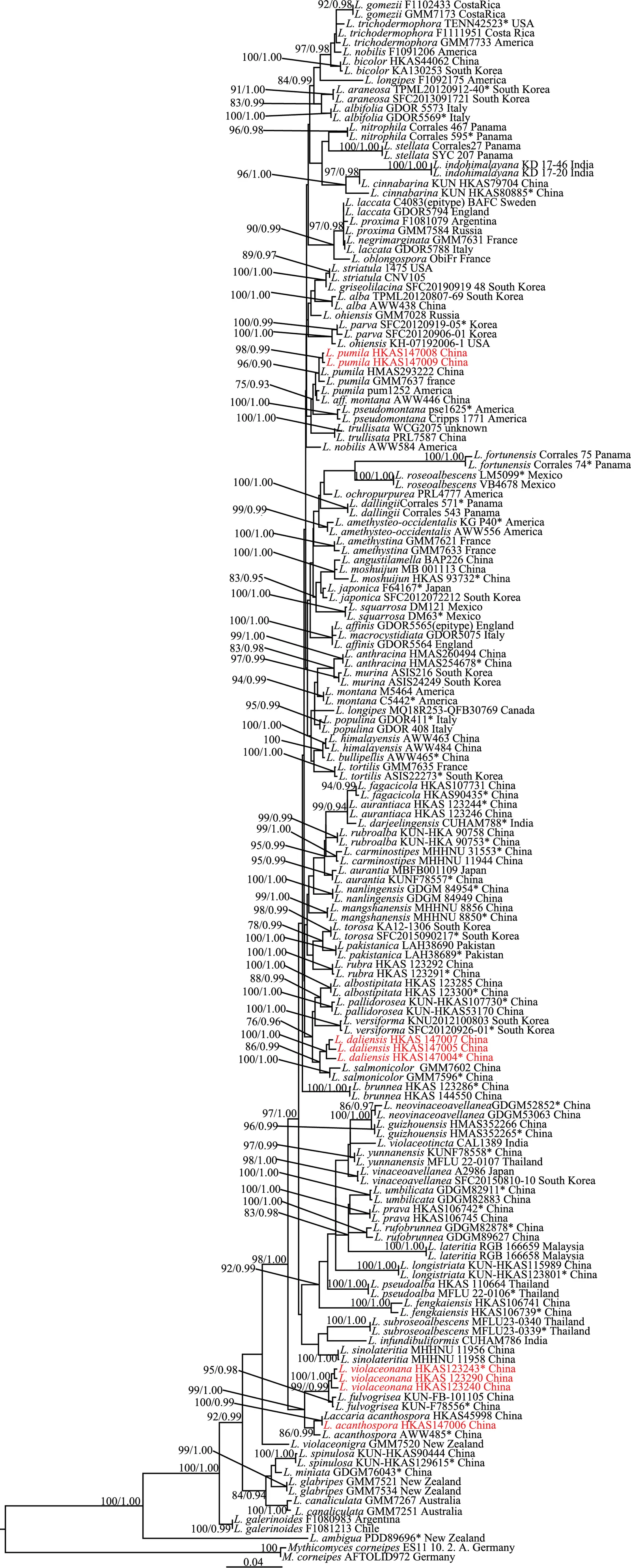
Maximum likelihood phylogeny based on nrITS, nrLSU, *rpb2*, and *tef1-α* sequence data used to identify species of *Laccaria*, rooted with *Mythicomyces corneipes*. MLBS values (≥75%) and PP values (≥0.90) are indicated above the branches or in front of the branch leading to each node. The specimens in this study are highlighted in red, and holotype specimens are marked with an asterisk (*).

Multilocus phylogenetic analysis showed that the nine *Laccaria* specimens clustered into four clades, each receiving strong support from both ML and Bayesian analyses (Fig. 1). Among them, two clades were identified as new species: *Laccaria daliensis* (100/1.00) and *L. violaceonana* (100/1.00); one clade was *L. acanthospora* A.W. Wilson & G.M. Muell., a newly recorded species in Yunnan (100/0.99); and one clade was the known species *L. pumila* Fayod (98/0.99). Thus, these species are treated in this paper. Support values such as “100/1.00” represent MLBS and Bayesian PP, respectively.

### Taxonomy

#### Laccaria daliensis

Taxon classificationFungiAgaricalesHydnangiaceae

G. Zhao, S.M. Tang & Z.L. Luo sp. nov.

ADD02B6B-8A2F-55BD-BB43-6B011C3235D8

Fungal Names: FN 572998

Figs 2, 3, 10d–f

##### Chinese name.

大理蜡蘑 (Da Li La Mo).

##### Diagnosis.

*Laccaria daliensis* is distinguished by its pale orange to light grayish orange basidiomata, subglobose to globose basidiospores measuring (6.5–)6.7–9.6(–10.1) × (6.2–)6.4–9.4(–9.8) μm, the presence of both narrowly utriform cheilocystidia and pleurocystidia, and hyphae of the pileipellis that are appressed and parallel.

##### Etymology.

The epithet “*daliensis*” refers to the type locality, Dali.

##### Holotype.

China • Yunnan Province, Dali Autonomous Prefecture, Shaxi Town, Shibao Mountain, 26°39'55.70"N, 100°24'32.02"E, elevation 2,500 m., 8 September 2024, G. Zhao, *2024090835* (HKAS147004).

##### Description.

**Basidiomata** medium size. **Pileus** 10–30 mm in diam., first broadly conical then plano-concave to concave, pale orange (#e2b78c), light orange (#e6c19b) to light grayish orange (#d9b38c), moderate orange (#d37445) at center, becoming paler towards the margin, without umbo when loss of moisture or with age becoming light grayish orange (#f8eac7), clearly striate on the surface, smooth, shiny, margin very thin, wavy; pileus context thin, below 1 mm, slightly desaturated orange (#d8bc99). **Lamellae** distant, ventricose, adnate with decurrent tooth, light grayish orange (#dfc8b9), 1–1.2 mm width; lamella edge even or entire, sometimes undate; lamellulae in 2–3 tiers. **Stipe** 20–50 × 2–6 mm, cylindrical, central or eccentric, equal with an enlarged base and nearly tapering upwards, light grayish orange (#f816cd) to slightly desaturated orange (#d5a27f), becoming light grayish orange (#f8eac7) after loss of moisture or with age, smooth, basal mycelium white (#ffffff); stipe context fistulose to broadly fistulose, dark moderate orange (#ae7857) to slightly desaturated orange (#d8ac8c). Taste and smell not observed.

**Basidia** 31–47 × 9–16 μm, av. 39 ± 3.9 × 13 ± 2.1 μm, clavate, mostly 4-spored, rarely 2-spored; sterigmata 4–9 × 2–4 μm, av. 6.5 ± 1.1 × 2.8 ± 0.5 μm. **Basidiospores** (excluding ornamentation) [168/3/3] (6.5–) 6.7–9.6 (–10.1) × (6.2–) 6.4–9.4 (–9.8) μm, av. 7.8 ± 0.6 × 7.7 ± 0.6 μm, *Q_m_* = 1–1.1, *Q_av_*_._ = 1.05 ± 0.01, subglobose to globose, hyaline, echinulate, spines 2–5 µm long, 1–2 µm wide at the base, crowded. **Cheilocystidia** 18–30 × 3–7 μm, av. 25 ± 2.5 × 4.7 ± 1.1 μm, narrowly clavate, thin-walled, colorless and hyaline, abundant. **Pleurocystidia** 13–22 × 3–6 μm, av. 17 ± 2.1 × 4 ± 0.7 μm, subclavate, narrowly clavate, flexuose, thin-walled, hyaline hyphae. The lamellar edge is predominantly composed of sterile basidia, which consist of clavate to cylindrically inflated cells (11–21 × 8–15 μm) that are thin-walled, hyaline, and similar in shape to basidioles. **Subhymenium** 15–30 μm thick, tightly interwoven, fusiform or irregular cells, 4–9 × 3–6 μm. **Lamellar trama** 70–100 μm thick, regular, composed of slightly thick-walled, filamentous hyphae 2–5 μm wide. **Pileipellis** 60–100 μm thick, colorless hyaline in 5% KOH, composed of appressed, parallel, simply septate, thin-walled, cylindrical, filamentous hyphae 5–14 μm wide, colorless and hyaline. **Stipitipellis** composed of appressed, parallel, simply septate, thick-walled hyphae 3–12 μm wide; stipe trama composed of longitudinally arranged, pastel red in 5% KOH, clavate terminal cells, infrequently branching, septate. **Caulocystidia** absent. Clamp connection present at some septa in pileipellis, lamellae, and stipitipellis.

**Figure 2.**
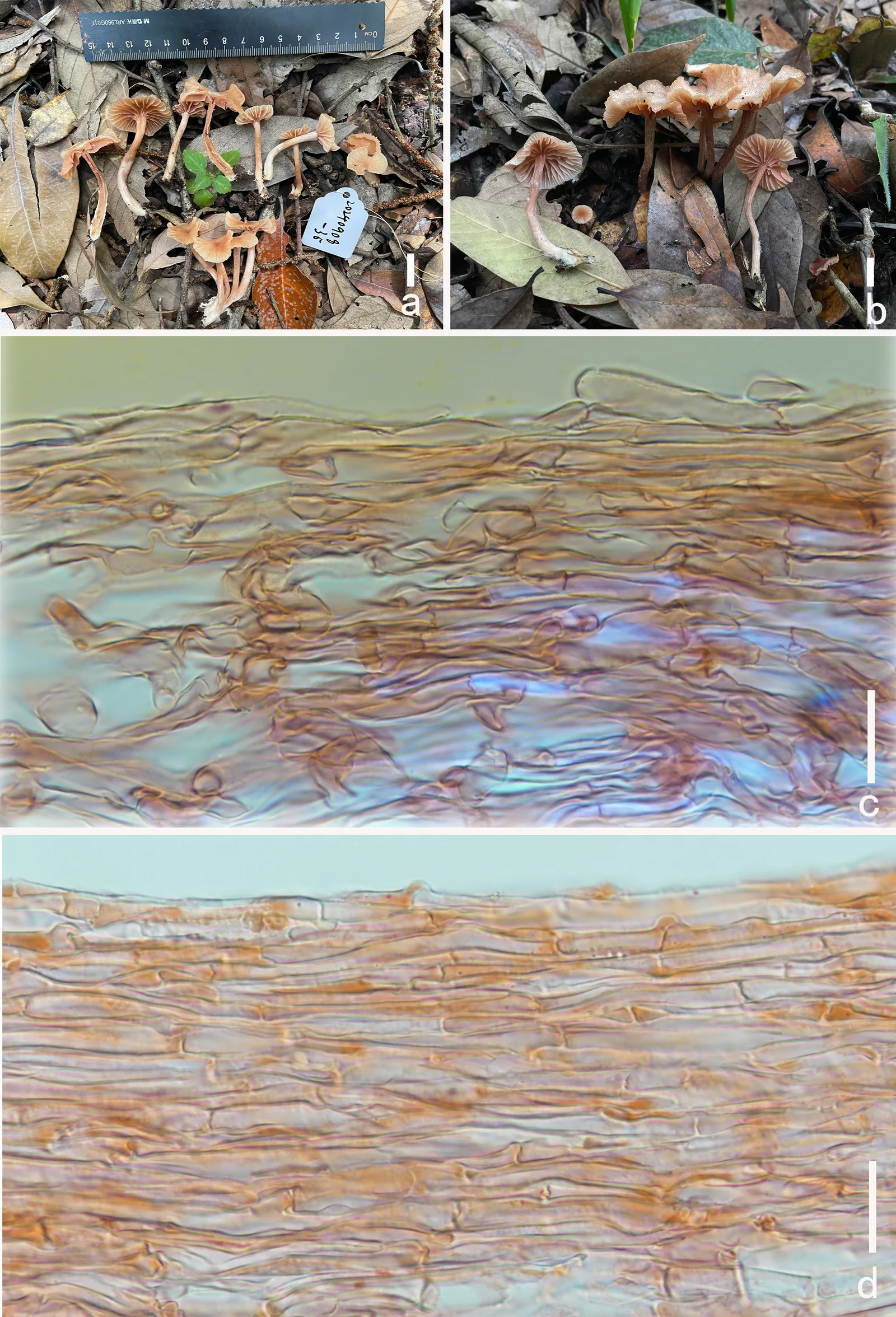
Fresh basidiomata of *Laccaria daliensis* (**a**. Holotype, HKAS 147004; **b**. HKAS 147007); **c**. Pileipellis; **d**. Stipitipellis. Scale bars: 1 cm (**a, b**); 20 μm (**c, d**). Photographs by Song-Ming Tang.

##### Habitat and phenology.

Growing scattered, gregarious, or caespitose in *Quercus* spp. forest lands.

##### Additional specimens examined (Paratype).

China • Yunnan Province, Dali Autonomous Prefecture, Shaxi Town, Shibao Mountain, 26°39'55.70"N, 99°84'32.02"E, ele. 2,500 m., 8 September 2024, G. Zhao, 2024090818 (HKAS 147005); ibid., 8 September 2024, G. Zhao, 2024090823 (HKAS 147007).

**Figure 3.**
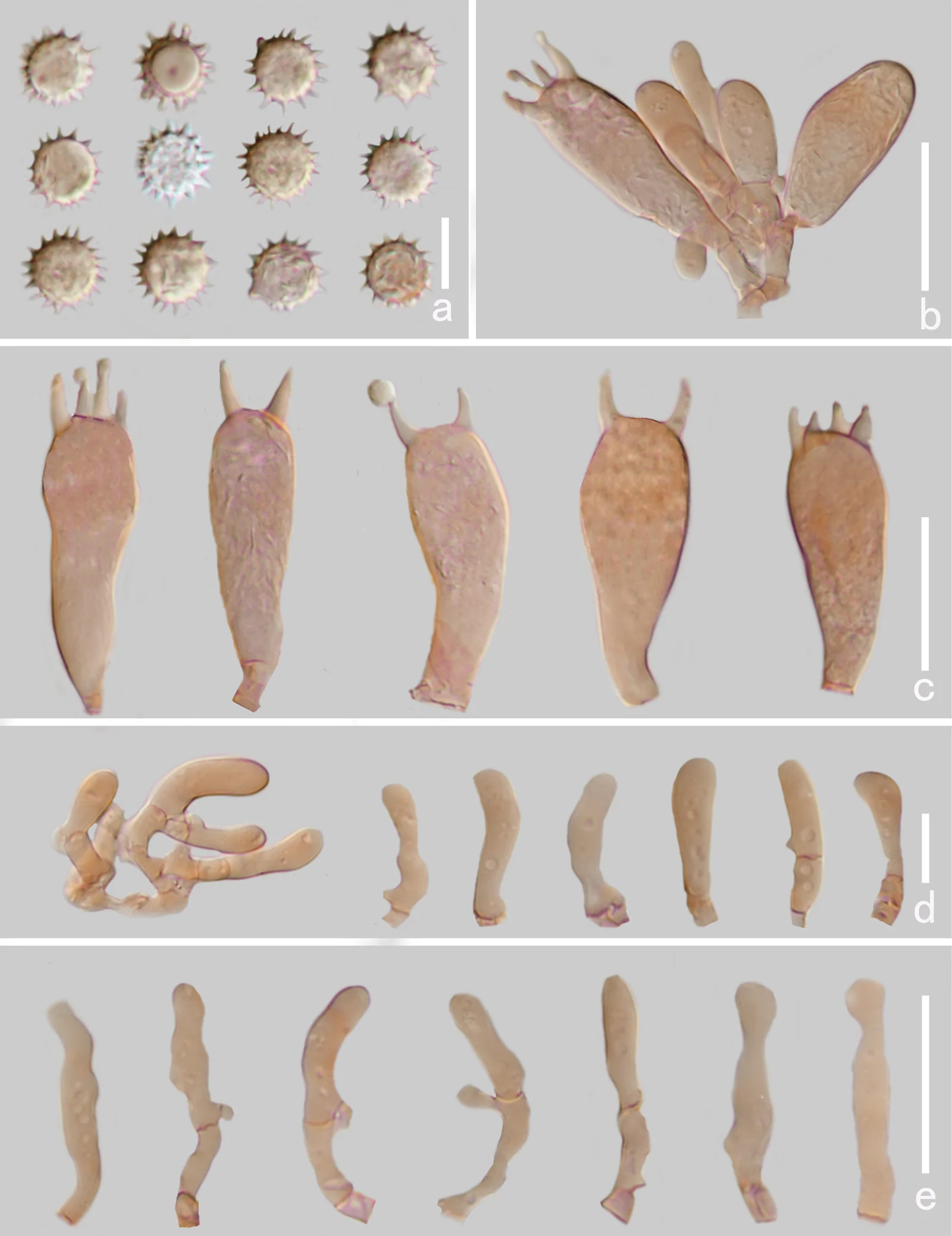
*Laccaria daliensis*: **a**. Basidiospores; **b**. Basidium and basidioles; **c**. Basidia; **d**. Pleurocystidia; **e**. Cheilocystidia. Scale bars: 10 μm (**a, d**); 20 μm (**b, c, e**).

##### Notes.

In the multi-locus phylogenetic analysis, *L. daliensis* is closely related to *L. salmonicolor* A.W. Wilson & G.M. Muell. However, *L. salmonicolor* is distinguished by its reddish-brown basidiomata that fade to pale buff, salmon-pink lamellae, and globose spores with medium-sized spines (2–5 × 1–2 μm vs. 1–3 × 1–2 μm) (Wilson et al. 2013).

Macroscopically, *Laccaria daliensis* exhibits similarities to several allied species—including *L. stellata* Popa & S.Y. Castillo, *L. himalayensis* A.W. Wilson & G.M. Muell., *L. aurantia* Popa, Rexer, Donges, Zhu L. Yang & G. Kost, *L. indohimalayana* K. Das, I. Bera & Vizzini, *L. umbilicata* Ming Zhang, *L. nitrophila* Corrales, Ovrebo, A.W. Wilson & G.M. Muell., *L. araneosa* H.J. Cho & Y.W. Lim, and *L. albifolia* Dovana & Para—particularly in its orange pileus coloration. Its overall appearance is especially similar to that of *L. stellata* and *L. umbilicata*, making them difficult to distinguish solely on macromorphology. Reliable differentiation, therefore, relies on micromorphological characteristics. Compared to *L. umbilicata*, *L. daliensis* has smaller basidiospores (minimum width < 8 μm) and distinctly smaller cheilocystidia (18–30 × 3–7 μm) and pleurocystidia (13–22 × 3–6 μm), whereas *L. umbilicata* possesses larger cheilocystidia (30–47 × 3–8 μm) and pileocystidia (28–38 × 4–6 μm) and lacks pleurocystidia (Zhang et al. 2023). When contrasted with *L. stellata*, *L. daliensis* shows a broader basidiospore width range (up to 9.8 μm) and distinctly produces both cheilocystidia and pleurocystidia, structures not reported in the original description of *L. stellata* (Popa et al. 2016). *L. daliensis* differs from *L. himalayensis* by having more prominent spore ornamentation, with longer and wider spines (2–5 μm long, 1–2 μm wide at the base vs. (0.5–)1–3 μm long and <1 μm wide in *L. himalayensis*), along with abundant cheilocystidia and pleurocystidia, which are absent in *L. himalayensis* (Wilson et al. 2013). Compared to *L. aurantia*, *L. daliensis* has distinctly smaller basidiomata, and its basidiospores, basidia, and cystidia are all consistently smaller (Popa et al. 2014). *Laccaria daliensis* can be distinguished from *L. indohimalayana* by its smaller basidiomata, broader basidiospores, and the presence of both cheilocystidia and pleurocystidia, structures that are entirely absent in the latter species (Wang et al. 2019).

Micromorphologically, *Laccaria daliensis* can be distinguished from *L. nitrophila* by its smaller basidiospores (av. 7.8 μm), longer and wider spore spines, and the presence of distinct cheilocystidia and pleurocystidia—structures completely absent in the latter (Corrales et al. 2020). Compared to *L. araneosa*, *L. daliensis* possesses a differentiated subhymenium, both cheilocystidia and pleurocystidia (lacking in *L. araneosa*), and longer, thicker spore spines (Cho et al. 2018). When contrasted with *L. albifolia*, the former features longer and thicker spines (2–5 μm long, 1–2 μm wide at the base), subglobose to globose basidiospores (av. 7.8 × 7.7 μm), the presence of distinct pleurocystidia, and a pileipellis composed of appressed, parallel hyphae. In contrast, *L. albifolia* is characterized by very short spines (up to 1.2 μm long, <1 μm wide at the base) and more variable basidiospore shapes (globose to ellipsoid, with a maximum *Q* value of 1.40) (Dovana et al. 2025).

#### Laccaria violaceonana

Taxon classificationFungiAgaricalesHydnangiaceae

G. Zhao, S.M. Tang & Z.L. Luo sp. nov.

3CF12F84-F737-5962-9E78-7AADEDA7E13F

Fungal Names: FN 572997

Figs 4, 5, 10a–c

##### Chinese name.

小紫蜡蘑 (Xiao Zi La Mo).

##### Diagnosis.

*Laccaria violaceonana* is characterized by medium-sized basidiomata, a convex pileus, a stipe with an enlarged base, deep purple, globose to subglobose basidiospores, and basidia that are mostly 4-spored.

##### Etymology.

The epithet “*violaceonana*” refers to the purple-violet coloration of its basidiomata and their smaller stature compared with *L. amethystina* Cooke.

##### Holotype.

China • Yunnan Province, Puer City, Ailao Mountain, 24°30'54.40"N, 100°16'42.08"E, ele. 2,300 m., 11 August 2020, S. M. Tang, 2020081105 (HKAS 123243).

##### Description.

**Basidiomata** medium size. **Pileus** 10–36 mm in diam., convex, becoming applanate to plano-concave, deep purple (#800080), smooth and translucent striate on the surface, a slightly depressed to depressed shape of center, becoming reflexed on the margin with age, margin undate, hygrophanous, and context thin, 0.6–1.5 mm. **Lamellae** distant, broad, segmentiform, subdecurrent, purple (#9400d3), concolorous to pileus, 1–2 mm in height, lamella edge even or entire; lamellulae in 1–2 tiers. **Stipe** 13–22 × 2–4 mm, cylindrical, central or eccentric, equal with an enlarged base, deep purple (#800080), concolorous with pileus, becoming dark brown (#8b4513) after loss of moisture or with age, smooth, basal mycelium white; stipe context broadly fistulose, deep purple (#800080). Taste and smell not observed.

**Figure 4.**
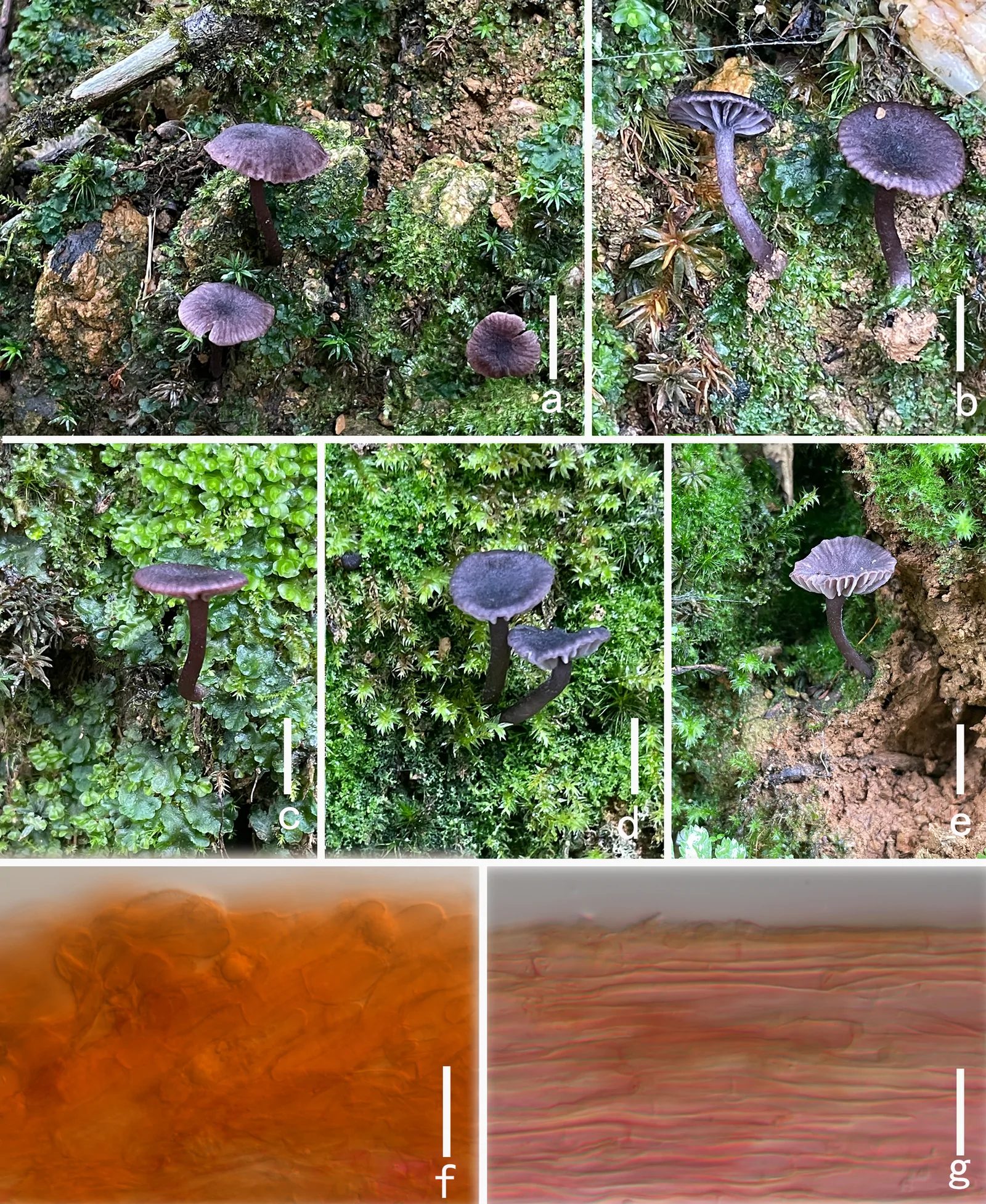
Fresh basidiomata of *Laccaria violaceonana*: **a**. Holotype, HKAS 123243; **b, c**. HKAS 123290; **d, e**. HKAS 123240; **f**. Pileipellis; **g**. Stipitipellis. Scale bars: 1 cm (**a–e**); 20 μm (**f, g**).

**Basidia** 29–47 × 10–16 µm, av. 39.7 ± 5.4 × 13.4 ± 1.6 μm, clavate, mostly 4-spored, rarely 2-spored, sterigmata 3–12 × 2–6 µm, av. 8.1 ± 1.7 × 2.9 ± 1.1 μm. **Basidiospores** (excluding ornamentation) [150/3/3] (5.5–) 6.0–9.6 (–9.9) × (5.1–) 5.4–9.0 (–9.5) µm, av. 7.5 ± 0.8 × 7.3 ± 0.8 μm, *Q_m_* = 1.0–1.12, *Q_av_*_._ = 1.03 ± 0.02, globose to subglobose, hyaline, echinulate, spines 1–4 µm long, 0.8–1.4 µm wide at the base, crowded. **Cheilocystidia** 26–76 × 4–13 µm, av. 48.9 ± 13.6 × 8.4 ± 2.3 μm, narrowly utriform, flexuose or mucronate, branches, thin-walled, colorless, and hyaline. **Pleurocystidia** 15–42 × 3–9 µm, av. 24.7 ± 7.5 × 5.5 ± 1.6 µm, narrowly clavate to subclavate, flexuose or mucronate, rarely branched, thin-walled, hyaline, abundant. **Lamellar trama** 113–120 µm thick, regular, composed of slightly thick-walled, filamentous hyphae 2–6 µm wide; lamellar edge more in several sterile basidia. **Subhymenium** 10–18 μm thick, tightly interwoven, fusiform or irregular cells, 8–11 × 2–7 μm. **Pileipellis** 25–50 µm thick, grayish, inflated hyaline in 5% KOH, composed of appressed, parallel, simply septate, thin-walled, cylindrical, filamentous hyphae 10–17 µm wide, colorless, and hyaline. **Stipitipellis** composed of appressed, parallel, simply septate, thick-walled, hyphae 4–8 µm wide; stipe trama is composed of longitudinally arranged, grayish in 5% KOH, clavate terminal cells, infrequently branching, septate, thick-walled, hyphae hyaline 3–10 µm wide. **Caulocystidia** absent. Clamp connection present at some septa in pileipellis, lamellae, and stipitipellis.

**Figure 5.**
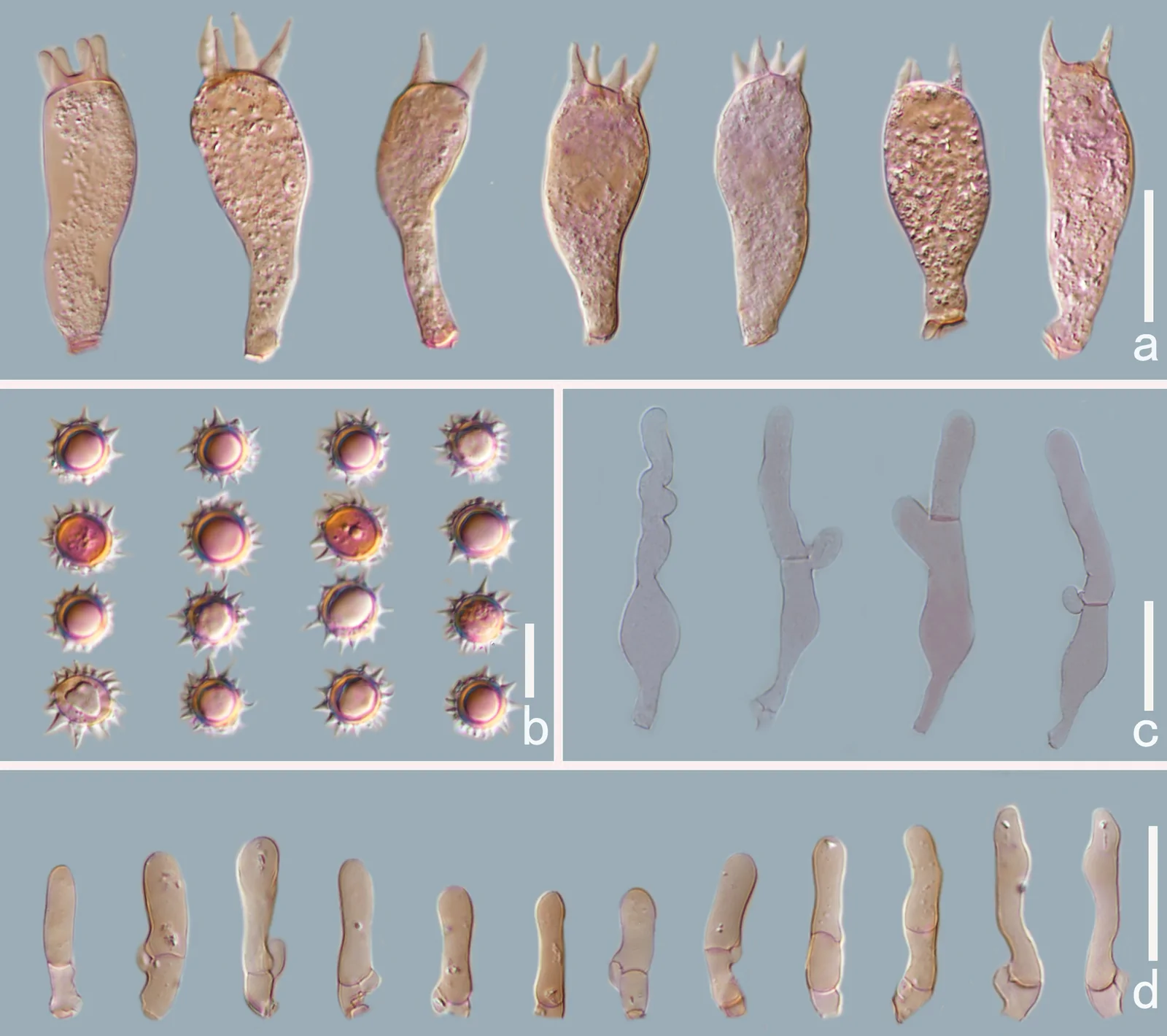
*Laccaria violaceonana*: **a**. Basidia; **b**. Basidiospores; **c**. Cheilocystidia; **d**. Pleurocystidia. Scale bars: 10 μm (**b**); 20 μm (**a, c, d**).

##### Habitat and phenology.

Scattered, gregarious, or caespitose on the edges of ditches, on sandy soils, and beside woods.

##### Additional specimens examined (Paratype).

China • Yunnan Province, Puer City, Ailao Mountain, 24°30'54.40"N, 100°16'42.08"E, ele. 2,300 m., 3 August 2020, S. M. Tang, 2020080301 (HKAS 123290); • ibid., 3 August 2020, S. M. Tang, 2020080303 (HKAS123240).

##### Notes.

In the multilocus phylogenetic analysis, *Laccaria violaceonana* is sister to *L. fulvogrisea* Popa, Rexer & G. Kost. However, *L. fulvogrisea* can be distinguished by its gray to brownish pileus and lamellae, larger stipe (30–70 × 3–5 mm) that is slightly darker gray than the pileus, larger basidia (40–50 × 10–15 µm), clavate and flexuous pleurocystidia, cheilocystidia (30–50 × 3–7 µm) ranging from clavate to subcapitate, and the absence of caulocystidia on the stipe surface (Popa et al. 2014). The ITS sequence divergence between *L. violaceonana* (HKAS 123243) and *L. fulvogrisea* (KUN F78556) is 2.03% (12/591, excluding gaps).

Macroscopically, *Laccaria violaceonana* shares purple basidiomata with *L. amethystina*, *L. amethysteo-occidentalis* G.M. Muell, *L. dallingii* Corrales, Ovrebo, A.W. Wilson & G.M. Muell, *L. japonica* Popa & K. Nara, *L. moshuijun*, and *L. violaceotincta* K.P.D. Latha, K.N.A. Raj & Manim but can be distinguished by a combination of micromorphological characteristics. Compared with *L. amethystina* and *L. amethysteo-occidentalis*, *L. violaceonana* possesses abundant pleurocystidia, which are absent in the latter two species. Additionally, its basidiospores (6.0–9.6 × 5.4–9.0 µm) are smaller than those of *L. amethystina* (7.5–9.5 × 7.5–9.5 µm), its basidia (29–47 × 10–16 µm) are smaller than those of *L. amethysteo-occidentalis* (34–56.5 × 9.7–14.7 µm), and its narrowly utriform cheilocystidia (26–76 × 4–13 µm) differ in shape from those of both species (Cooke 1884; Mueller 1984). In contrast to *L. dallingii*, *L. violaceonana* has pleurocystidia and narrowly utriform rather than simple cylindrical cheilocystidia and lacks the distinctive orange-pink KOH reaction of the hyphae observed in the latter (Corrales et al. 2020). It is distinguished from *L. japonica* and *L. moshuijun* primarily by its hyphal structure: *L. violaceonana* has appressed, parallel hyphae in both the pileipellis and stipitipellis, in contrast to the interwoven, radiating hyphae with intracellular pigmentation found in the latter two species. Furthermore, the spore spines of *L. violaceonana* (1–4 µm long) are longer than those of *L. japonica* (0.8–1 µm), and its cheilocystidia are narrowly utriform, differing from the cylindrical to clavate or apically branched or capitate forms found in *L. japonica* and *L. moshuijun* (Popa et al. 2017a, 2017b). The most notable distinction from *L. violaceotincta* is the presence of pleurocystidia in *L. violaceonana*, along with a broader spore size range (6.0–9.6 × 5.4–9.0 µm vs. 7–8 × 7–8 µm) (Latha et al. 2019).

#### Laccaria pumila

Taxon classificationFungiAgaricalesHydnangiaceae

Fayod, Annali R. Accad. Agric. Torion 35: 91 (1893)

2F36CE32-44BF-50D8-8159-2F1EF260B74A

Figs 6, 7, 10g–i

##### Chinese name.

矮蜡蘑 (Ai La Mo).

##### Description.

Basidiomata medium-sized. **Pileus** 10–56 mm in diameter, initially hemispherical to convex, becoming applanate with age, often plano-concave at maturity, occasionally with an uplifted margin; surface glabrous to finely fibrillose, becoming more distinctly fibrillose with age, distinctly striate to translucent-striate when fresh; hygrophanous, orange-brown (#a6532a) when fresh, fading rapidly to pale brown (#f2b790) and finally buff (#f0dcb4); margin crenate, orange-brown (#a6532a); context thin, 1–2 mm thick. **Lamellae** distant, segmentiform to subventricose, adnexed to subdecurrent, waxy, pinkish-flesh-colored (#d28c8c), 1–3 mm high; edge even or entire; lamellulae in 2–3 tiers. **Stipe** 20–76 × 3–6 mm, cylindrical, central, either nearly equal or slightly enlarged and subclavate toward the base, occasionally caespitose; surface dry, subglabrous to finely fibrillose, often faintly longitudinally striate, concolorous with the pileus; basal mycelium white; context broadly fistulose, orange-brown (#a6532a). Odor and taste not observed.

**Figure 6.**
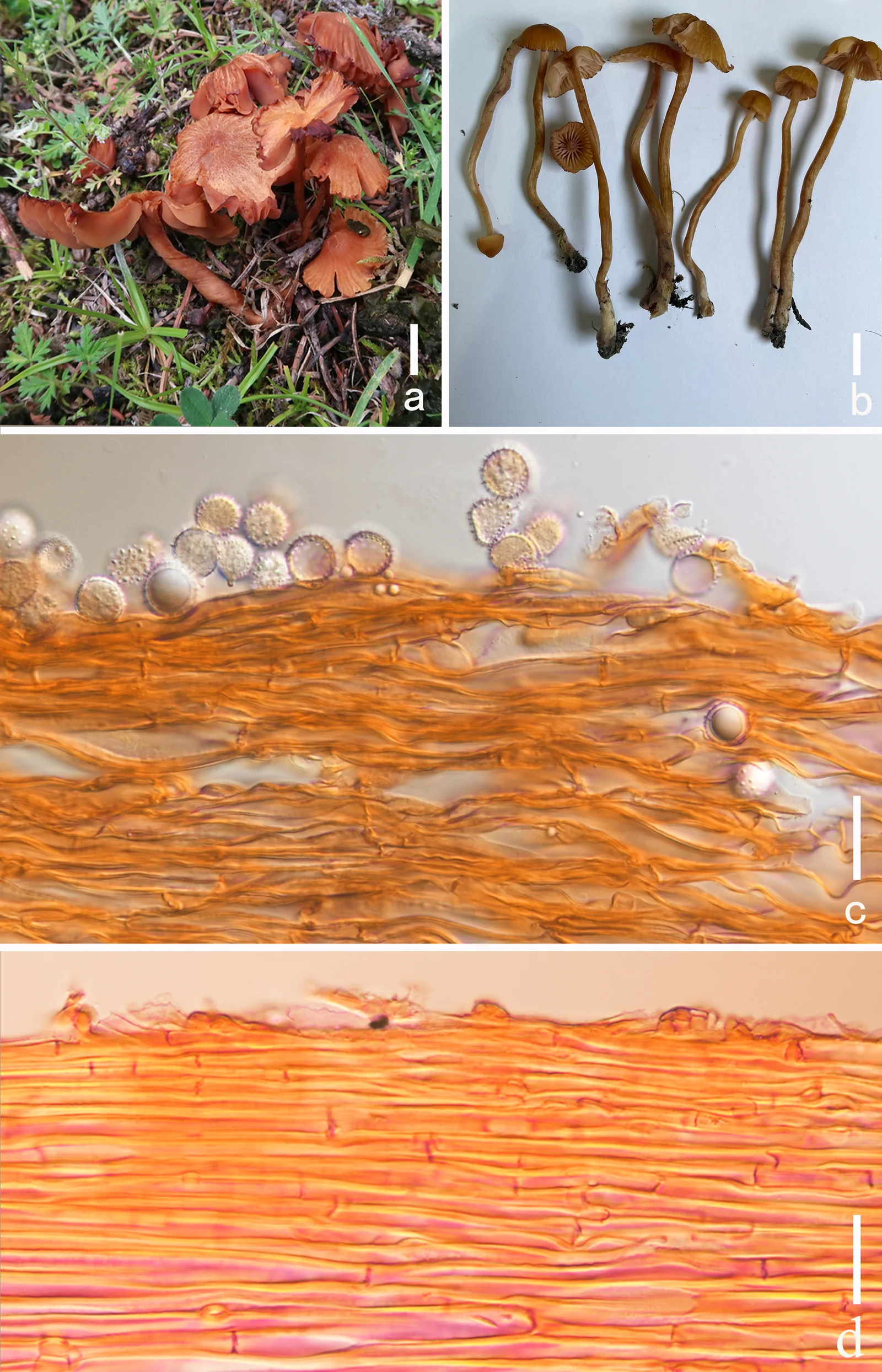
Fresh basidiomata of *Laccaria pumila*: **a**. HKAS 147008; **b**. HKAS 147009; **c**. Pileipellis; **d**. Stipitipellis. Scale bars: 1 cm (**a, b**); 20 μm (**c, d**).

**Basidia** 44–68 × 11–17 µm, av. 53.5 ± 5.9 × 14.1 ± 1.5 μm, clavate, 2–spored, sterigmata 6–14 µm × 3–5 µm, av. 10.1 ± 1.7 × 3.9 ± 1.5 μm. **Basidiospores** (excluding ornamentation) [100/2/2] (9.5–)10–13 (–14.1) × (8.9–) 9–12 (–13.5) µm, av. 11.4 ± 0.79 × 10.6 ± 0.98 μm, *Q_m_* = 1.02–1.13, *Q_av._* = 1.07 ± 0.30, subglobose to broadly ellipsoid, occasionally globose or ellipsoid, hyaline, echinulate, spines 1–2 µm long, 0.8–1.2 µm wide at the base, crowded. **Cheilocystidia** 18–39 × 4–8 µm, av. 29.5 ± 5.3 × 5.9 ± 0.9 μm, narrowly clavate, flexuose or mucronate, rarely branched, thin-walled, colorless, and hyaline, abundant. **Pleurocystidia** 15–33 × 3–7 µm, av. 23.6 ± 4.2 × 5.2 ± 1.1 µm, narrowly clavate to subclavate, flexuose or mucronate, rarely branched, thin-walled, hyaline. **Lamellar trama** 120–155 µm thick, regular, composed of slightly thick-walled, filamentous hyphae 3–8 µm wide; the lamellar edge is primarily composed of sterile basidia. **Subhymenium** 15–30 μm thick, tightly interwoven, fusiform or irregular cells, 3–8 × 3–7 μm. **Pileipellis** 100–130 µm thick, light orange hyaline in 5% KOH, composed of appressed, parallel, simply septate, thin-walled, cylindrical, filamentous hyphae 6–10 µm wide, colorless, and hyaline. **Stipitipellis** is composed of appressed, parallel, simply septate, thick-walled, hyphae 3–6 µm wide; stipe trama is composed of longitudinally arranged, pastel red in 5% KOH, clavate terminal cells, infrequently branching, septate, thick-walled, hyaline 3–9 µm wide. **Caulocystidia** absent. Clamp connection present at some septa in pileipellis, lamellae, and stipitipellis.

**Figure 7.**
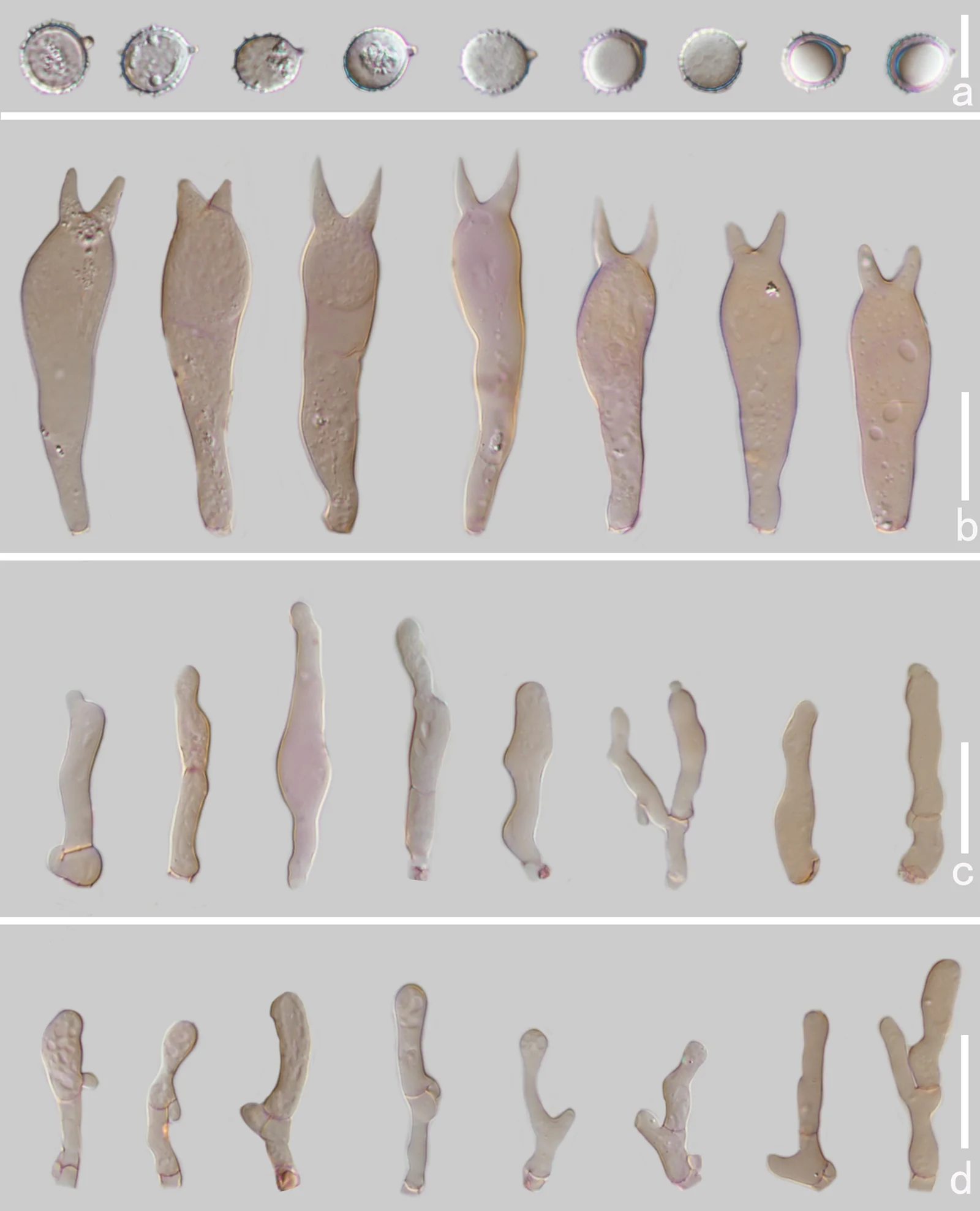
*Laccaria pumila*: **a**. Basidiospores; **b**. Basidia; **c**. Cheilocystidia; **d**. Pleurocystidia. Scale bars: 10 μm (**a**); 20 μm (**b–d**).

##### Habitat and phenology.

Scattered, gregarious, or caespitose on the edges of ditches, in grasslands, on sandy soils, and beside woods.

##### Additional specimens examined.

China • Xizang Autonomous Region, Linzhi City, 30°15'59.32"N, 96°38'54.57"E, ele. 3,700 m., 25 July 2024, S. M. Tang, 2024072521 (HKAS 147008); • ibid., 25 July 2024, S. M. Tang, 2024072522 (HKAS 147009).

##### Notes.

Following BLAST searches of NCBI GenBank, the closest matches to the ITS and LSU sequences of the collection (HKAS 147008) are *L. pumila* (specimen GMM7637, ITS 99.65% sequence identity; specimen AWW446, LSU 99.88% sequence identity). The morphology of the Xizang collections matches the description of *L. pumila*, originally described from France (Fayod 1893). However, unlike those described in previous studies, both the basidiomata (pileus 15–56 mm in diameter, stipe 20–76 × 3–6 mm) and basidia (44–68 × 11–17 μm) are larger, and pleurocystidia were also observed. Phylogenetically, the specimens clustered with *L. pumila* in the same clade. Therefore, based on both morphological and phylogenetic analyses, the two specimens are identified as *L. pumila*.

#### Laccaria acanthospora

Taxon classificationFungiAgaricalesHydnangiaceae

A.W. Wilson & G.M. Muell., in Wilson, Hosaka, Perry & Mueller, Mycoscience 54 (6): 412 (2013)

636EE179-984F-5391-BC2B-B5A902006EBF

Figs 8, 9, 10j–l

##### Chinese name.

刺孢蜡蘑 (Ci Bao La Mo).

##### Description.

Basidiomata small. **Pileus** 4–15 mm diameter, plano-concave to convex, with the center depressed to umbilicate, surface glabrous, at times weakly fibrillose, the center is moderate orange (#c17c55) and the edges are soft orange (#e99b60), margin striate, subhemispherical; pileus context thin, 1–2 mm. **Lamellae** adnate to adnate with decurrent tooth, broad, distant, lamella edge even or entire; lamellulae in 2–4 tiers, pale orange color than pileus (#ffceb3). **Stipe** 27–45 × 1–3 mm, cylindrical, fistulose, central or eccentric, fibrillose striate, base orange-pink (#fac794) with some brown streaks (#874a2b), transitioning to light orange (#e99b60); stipe context fistulose, soft orange (#e99b60), subbulbous at the base. Odor and taste not observed.

**Figure 8.**
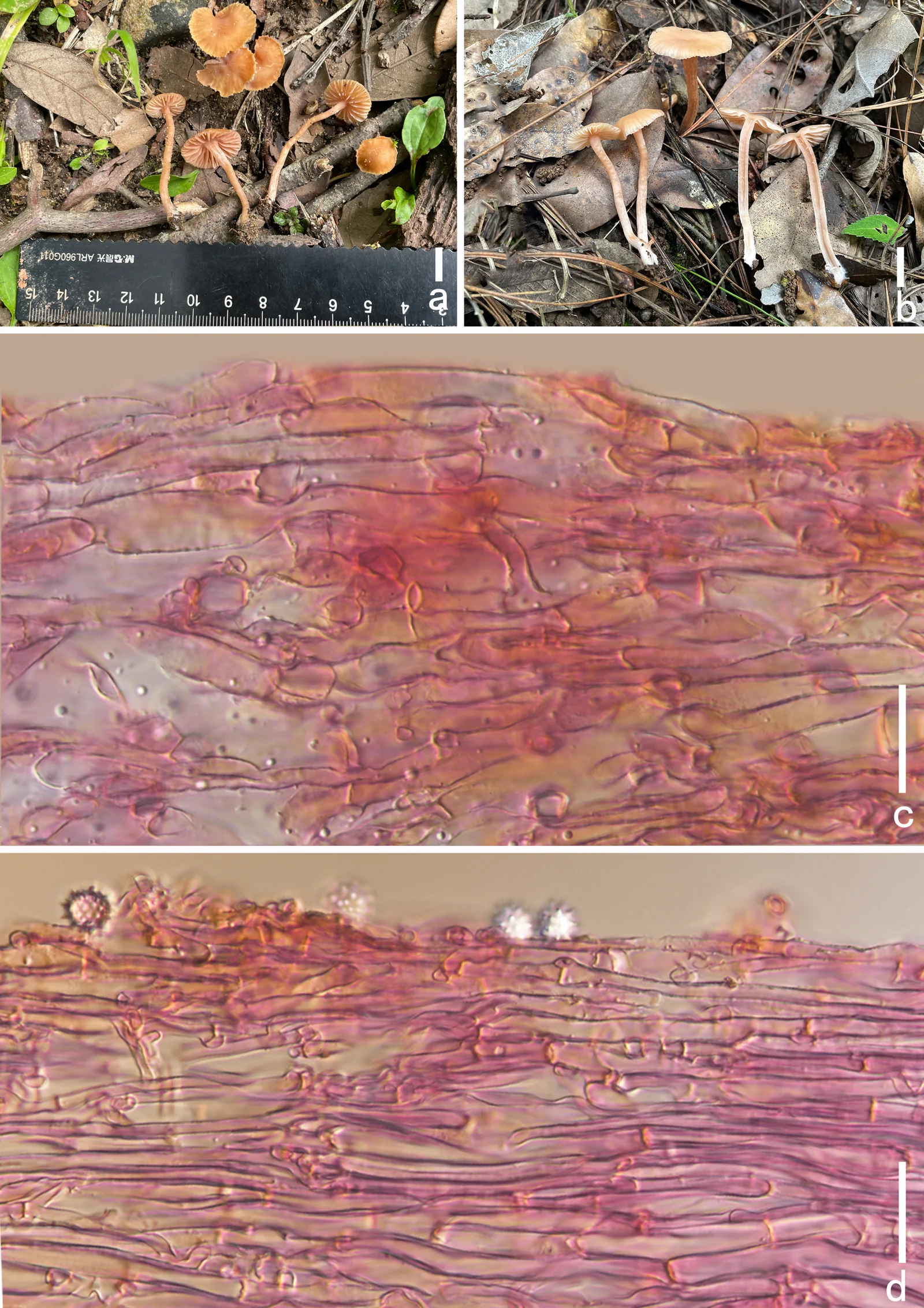
Fresh basidiomata of *Laccaria acanthospora*: **a, b**. HKAS 147006; **c**. Pileipellis; **d**. Stipitipellis. Scale bars: 1 cm (**a, b**); 20 μm (**c, d**). Photographs by Guo Zhao.

**Basidia** 35–50 × 9–15 μm, av. 38 ± 4.4 × 13 ± 1.6 μm, clavate, mostly 4-spored, rarely 2-spored; sterigmata 4–10 × 2–4 μm, av. 6.5 ± 1.2 × 2.7 ± 0.4 μm. **Basidiospores** (excluding ornamentation) [65/1/1] (6.6–) 7.1–10.8 (–11.1) × (6.4–) 7.0–10.6 (–10.9) μm, av. 9.1 ± 0.8 × 9.1 ± 0.8 μm, *Q_m_* = 1.0–1.1, *Q_av_*_._ = 1.0 ± 0.02, broadly ellipsoid to globose, strongly echinate; spines 2–6 × 1–2 μm, crowded, with acute hylar appendage. **Cheilocystidia** measuring 26–50 × 2–11 μm, av. 37.1 ± 7.7 × 6.5 ± 2.8 μm, polymorphic, predominantly subclavate to narrowly clavate or hyphoid in form, flexuose and irregular, with thin hyaline walls. **Pleurocystidia** 13–23 × 2–8 μm, av.17 ± 2.8 × 5 ± 1.1 μm, morphologically distinct from cheilocystidia, polymorphic, subclavate to narrowly clavate or hyphoid in form, flexuous or mucronate, occasionally exhibiting branching, with thin hyaline walls. **Lamellar trama** parallel to interwoven, composed of cylindrical hyphae 30–100 μm in diameter. **Subhymenium** 13–27 μm thick, tightly interwoven, fusiform or irregular cells, 3–7 × 2–5 μm. **Pileipellis** is a cutis composed of cylindrical hyphae 3–11 μm in diameter, interwoven and repent, with frequent branches. **Stipitipellis** composed of interwoven, cylindrical, clamped hyphae, 3–12 μm in diameter. **Caulocystidia** were not observed. Clamp connection present at some septa in pileipellis, lamellae, and stipitipellis.

**Figure 9.**
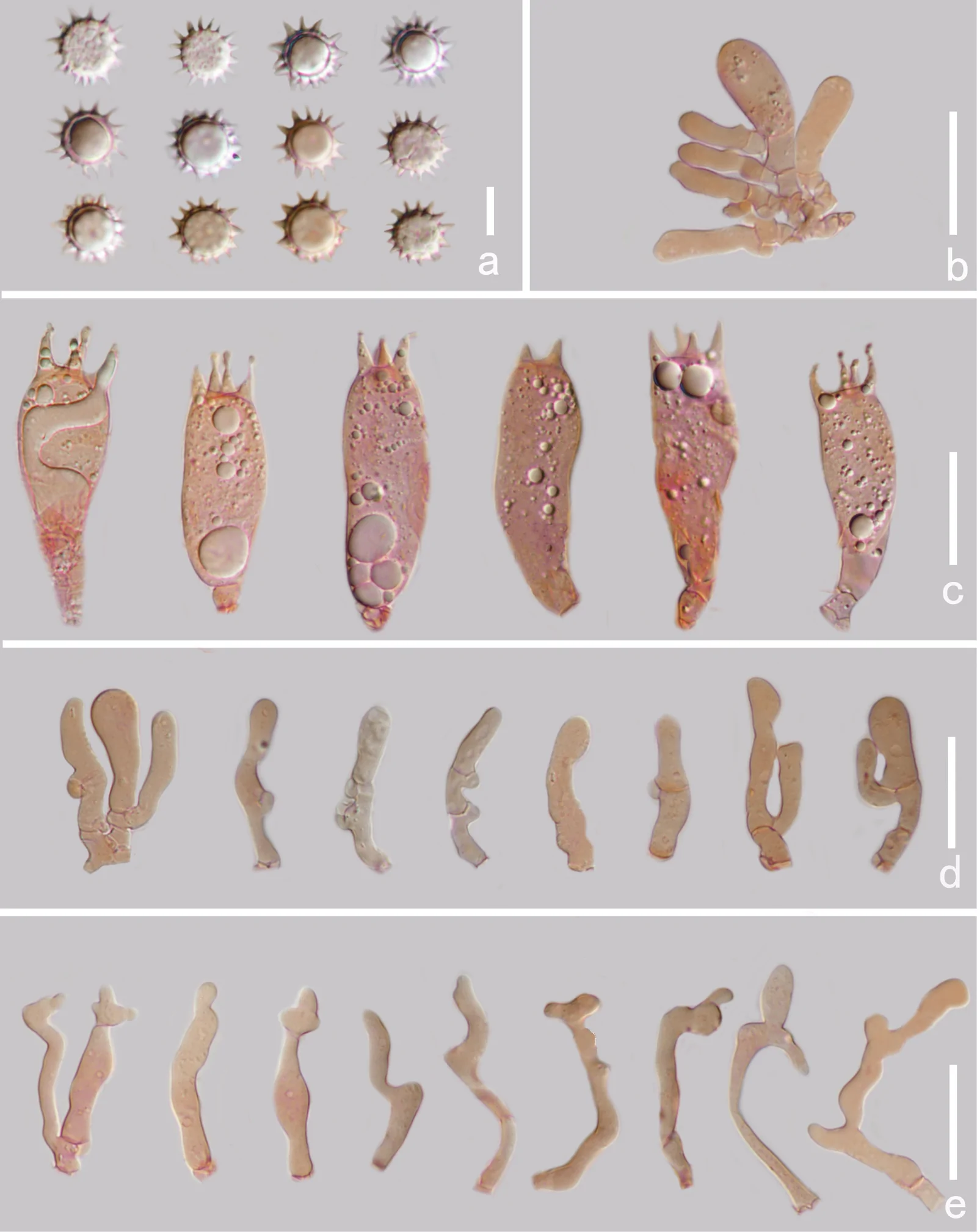
*Laccaria acanthospora*: **a**. Basidiospores; **b**. Basidium and basidioles; **c**. Basidia; **d**. Pleurocystidia; **e**. Cheilocystidia. Scale bars: 10 μm (**a**); 20 μm (**b–e**). Photographs by Guo Zhao.

##### Habitat and phenology.

Scattered, gregarious, or caespitose on the edges of ditches, in grasslands, on sandy soils, and beside woods.

##### Additional specimens examined.

China • Yunnan Province, Dali Autonomous Prefecture, Shaxi Town, Shibao Mountain, 26°39'55.70"N, 100°24'32.02"E, ele. 2,500 m., 8 September 2024, G. Zhao, 2024090827 (HKAS147006).

##### Notes.

Following BLAST searches of the NCBI GenBank database, the ITS and LSU sequences of the collected specimen (HKAS 147006) showed the highest similarity to *L. acanthospora* (99.63% sequence identity with specimen AWW485 for ITS and 99.88% with specimen HKAS45998 for LSU), and its morphological characteristics are consistent with the original description of *L. acanthospora* from Xizang by Wilson et al. (2013). Based on combined morphological and phylogenetic analyses, this specimen was identified as *L. acanthospora*. This species was originally described from specimens collected in Xizang, southwestern China, and this study represents a new collection record from Dali, Yunnan Province.

## Discussion

Based on morphological characteristics and multilocus molecular phylogenetic analyses, this study conducted a taxonomic investigation of the genus *Laccaria* in southwestern China, confirming two new species, *L. daliensis* and *L. violaceonana*, reporting a new record of *L. pumila* from Xizang and a new distribution record of *L. acanthospora* from Yunnan. These findings further enrich the species diversity records of the genus *Laccaria* in Asia and deepen understanding of its distribution patterns in southwestern China.

This study employed a four-locus concatenated analysis (nrITS–nrLSU–*rpb2*–*tef1-α*), which significantly improved the resolution and reliability of species delimitation within the genus *Laccaria*. Compared with previous studies that relied solely on nrITS sequences (Wilson et al. 2013; Popa et al. 2014, 2016; Luo et al. 2016; Latha et al. 2019; Cui et al. 2021), the multilocus phylogenetic approach effectively resolved the identification challenges posed by morphologically similar species within *Laccaria* and revealed the phylogenetic relationship between *L. daliensis* and its sister taxon, *L. salmonicolor*. Notably, although the *tef1-α* sequence of the *L. daliensis* specimen could not be successfully obtained, the combined analysis of nrITS, nrLSU, and *rpb2* data still provided sufficient molecular support for this new species. This study demonstrates that reliance on nrITS sequences alone may lead to underestimation or misdelimitation of species diversity, whereas the incorporation of protein-coding genes such as *rpb2* and *tef1-α* can provide more robust phylogenetic signals (Sheedy et al. 2013; Wilson et al. 2017; Cho et al. 2018).

*Laccaria violaceonana* is one of the few species with purple basidiomata within the genus and is sister to *L. fulvogrisea* in the phylogenetic tree. Purple basidiomata have been observed in multiple independent lineages within *Laccaria*, including *L. amethystina*, *L. amethysteo-occidentalis*, *L. japonica*, *L. moshuijun*, and *L. violaceotincta* (Mueller 1984; Popa et al. 2017a, 2017b; Latha et al. 2019), suggesting that this trait may have originated independently multiple times during the evolutionary history of the genus and may hold significant value for evolutionary biology research. Meanwhile, the discovery of *L. daliensis* further enriches the diversity of orange-toned *Laccaria* species. Orange-toned species are currently widely distributed across Asia, the Americas, and Oceania (Corrales et al. 2020; Zhang et al. 2023), indicating that this group possesses a transcontinental distribution pattern and potential for adaptive radiation, warranting further biogeographical investigation. The addition of new species and new distribution records also provides a critical taxonomic foundation for future studies on species differentiation, ecological adaptation, and the biogeographical history of the genus *Laccaria*.

The *Laccaria* specimens collected in this study were mainly distributed in western Yunnan and southeastern Xizang. As biodiversity hotspots in southwestern China, these regions provide rich ecological niches for *Laccaria* species due to their complex vertical climate zones and diverse vegetation types (Yang 2005; Wang et al. 2022). However, the current sampling scope remains relatively limited and fails to fully cover the potential distribution areas of the genus *Laccaria* across southwestern China and the broader Asian region. The new record of *L. pumila* from Xizang and the new distribution record of *L. acanthospora* from Yunnan indicate that with the expansion of the sampling range, the species diversity records of this genus in Asia are likely to increase further. Broader geographic sampling may reveal more cryptic species and novel biogeographic patterns (Sheedy et al. 2015). Therefore, future research should continue to expand the scope of field surveys to cover more under-investigated forest ecosystem types and combine multilocus sequence analyses to more comprehensively elucidate the biogeographic and evolutionary history of the genus *Laccaria* in Asia.

Based on morphological observations and multilocus phylogenetic analyses, this study systematically compared the four investigated species with their allied taxa. To facilitate differentiation and identification, Table 2 summarizes the main morphological characteristics of the *Laccaria* species involved in this study. The species exhibit clear and distinguishable differences in terms of pileus color, basidiospore size, and the presence or absence and morphology of cystidia. The new species *L. daliensis* is phylogenetically closely related to *L. salmonicolor*, but the former possesses pale orange to light grayish orange basidiomata and distinct cystidia, whereas the latter has reddish-brown basidiomata and lacks pleurocystidia (Wilson et al. 2013). The purple species *L. violaceonana* can be distinguished from other purple species such as *L. fulvogrisea*, *L. amethystina*, *L. japonica*, and *L. violaceotincta* by its smaller basidiomata, distinctive, narrowly utriform cheilocystidia, and appressed, parallel hyphae in both the pileipellis and stipitipellis. These integrated morphological and molecular identification methods have significantly improved the accuracy and reliability of species identification within the genus *Laccaria*, providing an effective approach to resolving taxonomic difficulties caused by morphological plasticity in this genus (Wang et al. 2022; Tang et al. 2025).

**Table 2.** Main morphological features of *Laccaria* species. Species newly described in this study are shown in bold.

**Species**	**Pileus**	**Cap color**	**Stipe size**	**Basidiospores size**	**Basidia size**	**Cheilocystidia size**	**Pleurocystidia size**	**reference**
***Laccaria acanthospora***	**4–15 mm**	**orange**	**27–45 × 1–3 mm**	**7**–**10.8 × 7**–**10.6 μm**	**35–50 × 9–15 μm**	**26–50 × 2–11 μm**	**13–23 × 2–8 μm**	**This study**
*L. alba*	10–35 mm	white to whitish	30–50 × 3–6 mm	7–9.5 × 7–9 μm	27–42 × 10–15 μm	20–48 × 4–6 μm	absent	Wang et al. 2004
*L. amethysteo-occidentalis*	10–65 mm	deep purple	18–115 × 3–12 mm	7.4–10.6 × 6.4–9 μm	34–57 × 9–15 μm	36–67 × 12–19 μm	absent	Mueller 1984
*L. ambigua*	8–10 mm	orange brown	15–17 × 4 mm	9–9.6 × 9–9.6 μm	32–40 × 9.5–11 μm	N/A	N/A	Wilson et al. 2017
*L. angustilamella*	20–30 mm	pinkish flesh to buff	40–60 × 3–5 mm	8.5–11.5 × 8–11.0 μm	31–46 × 10–18 μm	17–66 × 3–7 μm	absent	Wang et al. 2004
*L. anthracina*	25–65 mm	dark brownish gray to brownish black	40–120 × 3–8 mm	7–9.5 × 7–9 µm	36–53 × 9–14 μm	N/A	N/A	Wang et al. 2022
*L. amethystina*	10–52 mm	bright grayish purple to bufff	20–68 × 2–6 mm	7.5–9.5 × 7.5–9.5 µm	32–52 × 4.5–14 μm	33.5–86 × 2–12 μm	N/A	Cooke 1884
*L. araneosa*	10–18 mm	orange-brown to light brown	30–70 × 3–6 mm	8–9 × 7.5–9 μm	42–52 × 11–14 μm	absent	absent	Cho et al. 2018
*L. aurantia*	35–40 mm	orange	80 × 5 mm	9–10 × 8–10 μm	40–50 × 10–12 μm	25–40 × 5–10 μm	25–40 × 5–10 μm	Popa et al. 2014
*L. aurantiobrunneus*	20–35 mm	light brown to reddish brown	40–80 × 3–5 mm	7–8.5 × 6.5–8.0 μm	32–55 × 10–15 μm	absent	45–83 × 8–14 μm	Wang et al. 2019
*L. brownii*	15–27 mm	dark slightly desaturated orange	20–39 × 3–5 mm	6.5–8.4 × 6.1–7.7 µm	**21–40 × 8–14 µm**	13–20 × 3–7 µm	12–18 × 2–6 µm	Tang et al. 2025
*L. bicolor*	20–44 mm	ochraceous-rusty or slight tawny tinge	72–144 × 4–7 mm	7–9 × 6–7.5 μm	22–35 × 8**–**12 μm	24–52 × 2.5–7.5 μm	N/A	Orton 1960
*L. bullipellis*	22 mm	brown to orange-brown	71 × 4 mm	6–9 × 6–10 μm	40–50 × 10–11 μm	N/A	50–62 × 6–29 μm	Wilson et al. 2013
*L. bullulifera*	22 mm	brown to reddish-brown	71 × 4 mm	6–10 μm	36–48 × 10–11 μm	absent	20–46 × 4–7 μm	Singer and Moser 1965
*L. carminostipes*	15–45 mm	red-brown to orange-brown	70–90 × 3–7 mm	7–9 × 6.5–8 μm	38–46 × 10–13 μm	absent	absent	Xu et al. 2025
*L. cinnabarina*	10–90 mm	dark brown to orange-red	30–90 × 2–10 mm	7–9.5 × 7–9 μm	30–45 × 14–16 μm	absent	absent	Li et al. 2024
*L. canaliculata*	15–30 mm	flesh-colour to brownish	25–40 mm	9–10 × 9–10 μm	39–47 × 8–12 μm	N/A	N/A	Massee 1899
*L. cyanolamellata*	30–40 mm	grayish-blue	85–120 × 2–4 mm	11.4–14 × 11–15 μm	60–70 × 11–14 μm	N/A	N/A	Lechner et al. 2006
*L. dallingii*	12–22 mm	purple to brownish	12–30 × 1–5 mm	8–9 × 8–9 µm	31–45 × 11–13 µm	25–57 × 4–9 µm	N/A	Corrales et al. 2020
***L. daliensis***	**10–30 mm**	**pale orange, light orange**	**20–50 × 2–6 mm**	**6.7–9.6 × 6.4–9.4 μm**	**31–47 × 9–16 μm**	**18–30 × 3–7 μm**	**13–22 × 3–6 μm**	**This study**
*L. echinospora*	3–15 mm	pinkish brown to reddish brown	6–18 × 1–2 mm	16–21 × 2–3.5 μm	40–53 × 11–13 μm	N/A	N/A	Singer 1943
*L. fibrillosa*	5–35 mm	dark brown	20–70 × 1.5–3 mm	1.5–2.5 × 10–12.5 μm	35–53 × 10–14 μm	N/A	N/A	McNabb 1972
*L. friesii*	17–90 mm	slightly dark brown dusty brown	62–85 × 5–13 mm	9.8–12.4 × 9.8–12 μm	49–71 × 10–14 μm	32–59 × 14–17 μm	absent	Choudhary et al. 2025
*L. fulvogrisea*	< 30 mm	gray and with a violet to reddish brown	30–70 × 3–5 mm	8–10 × 8–11 μm	40–50 × 10–15 μm	30–50 × 3–7 μm	30–50 × 3–7 μm	Popa et al. 2014
*L. fagacicola*	20–45 mm	brownish orange to brownish	45–100 × 5–6 mm	7–9(10) × 6.5–8 μm	45–60 × 9–12 μm	15–50 × 3–8 μm	absent	Cui et al. 2021
*L. fortunensis*	8–15 mm	light orange-tan to brownish orange	20–42 × 3–5 mm	7–8 × 7–8 μm	33–49 × 8–11 μm	absent	absent	Corrales et al. 2020
*L. fengkaiensis*	50–90 mm	orange white, pale orange, light orange	60–100 × 6–9 mm	5.2–6.3 × 5.1–6.3 μm	30–45 × 6–8.5 μm	N/A	absent	Li 2020
*L. griseolilacina*	20–35 mm	light grayish lavender or orange-brown	40–60 × 0.3–0.8 mm	8.0–10.8 × 8–11 μm	36–42 × 10–13 μm	absent	20–31 × 3–8 μm	Cho et al. 2020
*L. glabripes*	5–25 mm	flesh pink to reddish brown	25–60 × 1.5–3.5 mm	8–11.5 × 1–1.7 μm	32–48 × 7–10.5 μm	N/A	N/A	McNabb 1972
*L. gomezii*	10–38 mm	dark violet to dark reddish	44–112 × 2–9 mm	7.5–9.3 × 6.8–8.3 μm	27–43 × 6.5–14 μm	64–86 × 6–11 μm	Absent	Mueller and Singer 1988
*L. guizhouensis*	15–63 mm	flesh-coloured, brownish-white, brown	45–86 × 2–8 mm	7–9 × 6–8.5 μm	27–40 × 8–11 μm	20–35 × 3–6 μm	27–46 × 3–7 μm	Zhang et al. 2024
*L. himalayensis*	6–34 mm	brown to orange-pink	10–50 × 2–5 mm	6.5–10 μm	34–46 × 10–14 μm	N/A	N/A	Wilson et al. 2013
*L. infundibuliformis*	8–27 mm	pinkish white to reddish gray pastel red	35 × 2 mm	5.2–11.8 × 5–12 µm	29–50 × 6–9 µm	N/A	N/A	Thapa et al. 2024
*L. indohimalayana*	40–95 mm	light orange	80–150 × 12–65 mm	7.6–8.3 × 6.6–7.2 μm	25–44 × 9–11.5 μm	Absent	Absent	Wang et al. 2019
*L. japonica*	10–30 mm	violet to purple	30–80 × 5 mm	9–10 × 7–9 μm	30–40 × 10–12 μm	30–50 × 3–7 μm	30–50 × 3–7 μm	Popa et al. 2017a
*L. longistriata*	5–45 mm	brownish, flesh-colored to brownish	40–60 × 3–8 mm	6.5–8 × 6–8 μm	27–42 × 10–13 μm	30–60 × 4–8 μm	Absent	Li et al. 2024
*L. laccata*	20–50 mm	vinaceous-pink or buff-pink	50–100 × 5–10 mm	8.5–10.5 × 7.5–9 μm	27–55.5 × 6–13.5	25–70 × 2–7.5	N/A	Imai 1938
*L. lilacina*	40–55 mm	fawn	50–100 × 3–5 mm	8.5–11 × 1.2–1.8 μm	35–50 × 8–11 μm	N/A	N/A	McNabb 1972
*L. longipes*	11–55 mm	orange brown to buff	67–138 × 3–9 mm	7–8.5 × 6–7.8 μm	28–44 × 7–10 μm	Absent	Absent	Mueller 1991
*L. mangshanensis*	10–30 mm	reddish-brown to rosy	20–70 × 2–5 mm	7–8.5 × 6.5–8.5 μm	38–47 × 10–13 μm	25–55 × 3–5 μm	Absent	Xu et al. 2025
*L. macrobasidia*	15–45 mm	orange-brown or light-brown	10–50 × 0.5–1.0 mm	8.7–11.4 × 7.9–10 μm	52–66 × 11–13 μm	Absent	19–40 × 4–7 μm	Cho et al. 2020
*L. miniata*	10–15 mm	red, reddish orange to yellowish red	35–60 × 1.5–2 mm	8–10.5 × 8–10 μm	40–58 × 8–15 μm	Absent	Absent	Zhang et al. 2023
*L. macrocystidiata*	5–25 mm	flesh coloured	25–90 × 2–5 mm	8 × 8 μm	40–45 × 11–15 μm	35–80 × 8–10 μm	N/A	Sesli et al. (2013)
*L. maritima*	45 mm	dark reddish brown to reddish orange	5–26 × 2–5 mm	14.5–17 × 7.5–9.5 μm	37–60 × 9–16 μm	N/A	N/A	Mueller 1991
*L. masonii*	5–35 mm	violaceous to buff tints	100 × 1–2.5 mm	2–3.5 × 10.5–14.5 μm	30–41 × 7.5–12 μm	N/A	N/A	McNabb 1972
*L. montana*	7–35 mm	orange brown to red brown	10–28 × 2–4 mm	8–11 × 8–9.5 μm	N/A	N/A	N/A	Osmundson et al. 2005
*L. moshuijun*	<30 mm	violet to bluish	100 × 3–5 mm	8–9 × 9–10 μm	30–40 × 10–12 μm	30–50 × 5–8 μm	30–50 × 3–6 μm	Popa et al. 2017a
*L. murina*	10–15 mm	reddish brown to reddish orange brown	15–25 × 1–3 mm	7.5–10 μm	13–17 × 7–10 μm	N/A	N/A	Imai 1938
*L. nanlingensis*	30–55 mm	orange, reddish orange, orange-red	25–70 × 2–5 mm	6.5–7.5 × 6–7 µm	35–48 × 8–12 µm	40–60 × 4–6 µm	Absent	Zhang et al. 2023
*L. neovinaceoavellanea*	15–40 mm	pastel pink, purplish pink to pale violet	30–70 × 2–5 mm	7–8 × 7–8 µm	30–50 × 10–14 µm	25–50 × 4–8 µm	Absent	Zhang et al. 2023
*L. negrimarginata*	5–15 mm	dark blackish brown to dark brown	30–70 × 2–5 mm	7–10 × 6–10 μm	30–45 × 10–15 μm	N/A	N/A	Wilson et al. 2013
*L. nobilis*	24–77 mm	brown	26–100 × 4–10 mm	7.4–9.7 × 6.4–8.7 μm	32.2–55 × 7–14 μm	Absent	Absent	Mueller 1984
*L. nitrophila*	7–20 mm	orange-brown, brownish orange	15–28 × 2–3 mm	8–9 × 8–9.5 μm	35–50 × 12–13 μm	Absent	Absent	Corrales et al. 2020
*L. oblongospora*	12–59 mm	brownish orange	20–60 × 2–12 mm	7.4–10 × 5–7 μm	24–35 × 6.4–10 μm	31.5–53 × 2.8–7 μm	Absent	Mueller 1984
*L. orangei*	18–32 mm	soft orange	46–55 × 3.4–5.5 mm	5.8–8.0 × 5.5–7.3 µm	30–40 × 7–10 µm	20–49 × 4–5 µm	15–30 × 5–7 µm	Tang et al. 2025
*L. ochropurpurea*	50 mm	grayish-pale alutaceous	55 × 15 mm	8–10 μm	N/A	N/A	N/A	Berkeley 1845
*L. ohiensis*	10–35 mm	reddish brown to dark reddish brown	25–70 × 2–5 mm	9.5–12.5 μm	32–45 × 7–10.5 μm	N/A	N/A	McNabb 1972
*L. olivaceogrisea*	9–32 mm	olivaceous beige to brownish	23–45 × 2.5–6 mm	7.5–9 × 8–9 μm	39–47 × 10–11 μm	Absent	Absent	Vellinga 1987
*L. pakistanica*	58–63 mm	dark brown, light reddish brown	84–89 × 6–10 mm	6.7–7.3 × 6.6–7.1 μm	38–47 × 8–16 µm	16–28 × 7–11 µm	18–31 × 5–9 µm	Rafique et al. 2025
*L. pallida*	15–45 mm	grayish red to pale red or pinkish white	48 × 4 mm	5.9–6.2 × 5.9–6.2 μm	29–37 × 11–14 µm	N/A	N/A	Thapa et al. 2024
*L. populina*	15–35 mm	orange to brownish-red	10–60 × 2–6 mm	7.2–8.8 × 7.1–8.8 µm	40–50 × 11–13 µm	N/A	40–50 × 3–7 µm	Dovana et al. 2021
*L. pseudoalba*	9–15 mm	pale orange, orange-white	28–41 × 1.8–2.7 mm	7.1–11.0 × 7–10.4 μm	29–38 × 9–13 μm	20–31 × 6–9 μm	15–31 × 6–8 μm	Tang et al. 2024
*L. pallidorosea*	10–25 mm	brownish to pinkish	22–40 × 2–4 mm	7–9 × 6.5–8.5 μm	25–40 × 10–15 μm	25–40 × 3–8 μm	Absent	Cui et al. 2021
*L. prava*	30–75 mm	pastel red, pale red, to reddish white	50–90 × 5–8 mm	6.5–7.5 × 7–8 μm	28–36 × 8–11 μm	15–23 × 4–7 μm	absent	Li 2020
*L. parva*	5–25 mm	bright brown-reddish brown	20–40 × 3–5 mm	8–10 × 8.5–10 um	44–56 × 12–15 μm	19–40 × 3.5–6 μm	25–33 × 4.5–5.5 μm	Cho et al. 2018
*L. proxima*	20–55 mm	brick red to reddish brown	30–90 × 3–6 mm	8–10.5 × 7.5–9.5 um	32–45 × 8–11 μm	N/A	N/A	McNabb 1972
*L. pruinosipes*	7–23 mm	dark flesh-coloured brown	55 × 2.5 mm	8–9 × 8–10 μm	N/A	22–32 × 6–9 μm	N/A	Pázmány 1994
*L. pseudomontana*	4–10 mm	dark orange to red brown	10–15 × 1–2 mm	7.5–11 × 6.5–10 μm	32–38 × 10–13 μm	Absent	Absent	Osmundson et al. 2005
***L. pumila***	**10–56 mm**	**pale orange-brown or red-brown**	**20–76 × 3–6 mm**	**10–13 × 9–12 µm**	**44–68 × 11–17 µm**	**18–39 × 4–8 µm**	**15–33 × 3–7 µm**	**This study**
*L. rubra*	18–22 mm	soft orange	34–49 × 3–5.5 mm	7–10.3 × 7–9.4 µm	33–39 × 10–14 µm	14–35 × 4–7 µm	18–48 × 3–5 µm	Tang et al. 2025
*L. roseoalbescens*	7–29 mm	pale pinkish rose to slight buff	12–50 × 1–4 mm	6.5–9 × 6.5–9 μm	32–58 × 8–17 μm	Absent	Absent	Montoya et al. 2015
*L. rufobrunnea*	12–35 mm	brownish orange to brownish red	20–40 × 3–5 mm	8–9 × 7–8 µm	40–54 × 10–15 µm	35–50 × 3–7 µm	Absent	Zhang et al. 2023
*L. rubroalba*	22–40 mm	reddish white	20–31 × 2–4 mm	6–9 × 5–7 μm	28–56 × 9–10 μm	12–26 × 5–9 μm	25–40 × 4–6 μm	Luo et al. 2016
*L. subroseoalbescens*	2–8 mm	light yellow, pale orange grayish orange	5–13 × 0.8–2 mm	7–8.9 × 6.8–9.0 μm,	30–46 × 8–14 μm	23–37 × 4–8 μm	36–59 × 5–8 μm	Tang et al. 2024
*L. stipalba*	18–42 mm	dark orange, light grayish pink	46–66 × 2–6 mm	5.8–8.4 × 5.5–8.1 µm	29–35 × 10–12 µm	20–30 × 4–7 µm	12–26 × 2–7 µm	Tang et al. 2025
*L. sinolateritia*	10–40 mm	brownish red	35–50 × 1–3 mm,	7.5–10 × 7.5–10 μm	40–58 × 12–14 μm	Absent	Absent	Xu et al. 2025
*L. spinulosa*	8–25 mm	brownish orange to brown	15–30 × 2–5 mm,	9–11 × 9–10.5 μm	43–53 × 10–14 μm	Absent	Absent	Li et al. 2024
*L. salmonicolor*	10–35 mm	reddish-brown to pale brown-buff	20–65 × 3 mm	7.5–10 μm	39–52 × 12–14 μm	N/A	Absent	Wilson et al. 2013
*L. squarrosa*	10–82 mm	pinkish to brownish orange	50–155 × 5–9 mm	7–10 × 7–10.5 um	35–66 × 10–15 µm	14–40 × 2–5 µm	20–38 × 3–6 µm	Ramos et al. 2017
*L. stellata*	14–22 mm	pinkish orange to flesh colored	15–25 × 2 mm	7.5–8.5 × 7.5–9 μm	37–43 × 9–12 µm	25–40 × 5–7 µm	25–40 × 5–7 µm	Popa et al. 2016
*L. tetraspora*	5–20 mm	reddish brown	10–30 × 1.5–2.5 mm	7.5–14 × 7–13.5 μm	40–50 × 11.5 μm	N/A	N/A	Singer 1947
*L. torosa*	10–70 mm	orange-brown to brown	35–95 × 5–13 mm	8–8.3–9 × 8–9.5 μm	39–47 × 12–17 μm	54–94 × 5–8.5 μm	55–75 × 7–13 μm	Cho et al. 2018
*L. tortilis*	5–10 cm	flesh colour	8–12 × 1–5 mm	12–16 μm	N/A	N/A	N/A	Singer 1946
*L. trichodermophora*	9–66 mm	brownish orange to reddish brown	22–125 × 2–11 mm	7.4–9.2 × 6.4–8.3 μm	25.8–46 × 7–12.4 μm	Absent	Absent	Mueller 1984
*L. trullisata*	25–50 mm	reddish flesh	25–70 × 5–8 mm	15–20 × 8–9 μm	N/A	N/A	N/A	Peck 1912
*L. umbilicata*	10–28 mm	pale yellow, pale orange to light orange	20–40 × 2–3 mm	8–10 × 8–10 µm	40–45 × 10–14 µm	30–47 × 3–8 µm	Absent	Zhang et al. 2023
*L. versiforma*	10–35 mm	pale brown-orange buff	30–35 × 2–4 mm	7.5–10 × 7.5–9.5 μm	41–55 × 10–14 μm	42–54 × 6–8 μm	42–65 × 6.5–8.5 μm	Cho et al. 2018
*L. vinaceobrunnea*	7–25 mm	brown to reddish brown	7–56 × 2–7 mm	7.4–10 × 6.4–9.2 μm	33–60 × 8.5–9.2 μm	31–92 × 5.5–11 μm	Absent	Mueller 1984
*L. violaceoniger*	5–35 mm	dark brown	30–60 × 3–6 mm	8–10 × 0.7–1.5 µm	32–42 × 7.5–10.5 µm	N/A	N/A	McNabb 1972
*L. vinaceoavellanea*	17–80 mm	pale flesh-brown	30–90 × 3–15 mm	6.8 × 7.8 µm	21.9–36 × 8.5–12 µm	N/A	17–22 × 6–7.3 μm	Hongo 1971
***L. violaceonana***	**10–36 mm**	**deep purple**	**13–22 × 2–4 mm**	**6.0–9.6 × 5.4–9.0 µm**	**29–47 × 10–16 µm**	**26–76 × 4–13 µm**	15–42 × 3–9 µm	**This study**
*L. violaceotincta*	4–38 mm	dark brown to reddish brown	16–50 × 1.5–4 mm	7–8 × 7–8 µm	31–47 × 10–13 µm	19–104 × 4–10 µm	absent	Latha et al. 2019
*L. yunnanensis*	6–10 cm	brownish to flesh-coloured	30–100 × 3–15 mm	8–9 × 8–10 μm	45–50 × 9–10 μm	30–50 × 4–6 μm	55–65 × 15–25 μm	Popa et al. 2014

Annotation: “N/A” refers to not mentioned in the original description.

**Figure 10.**
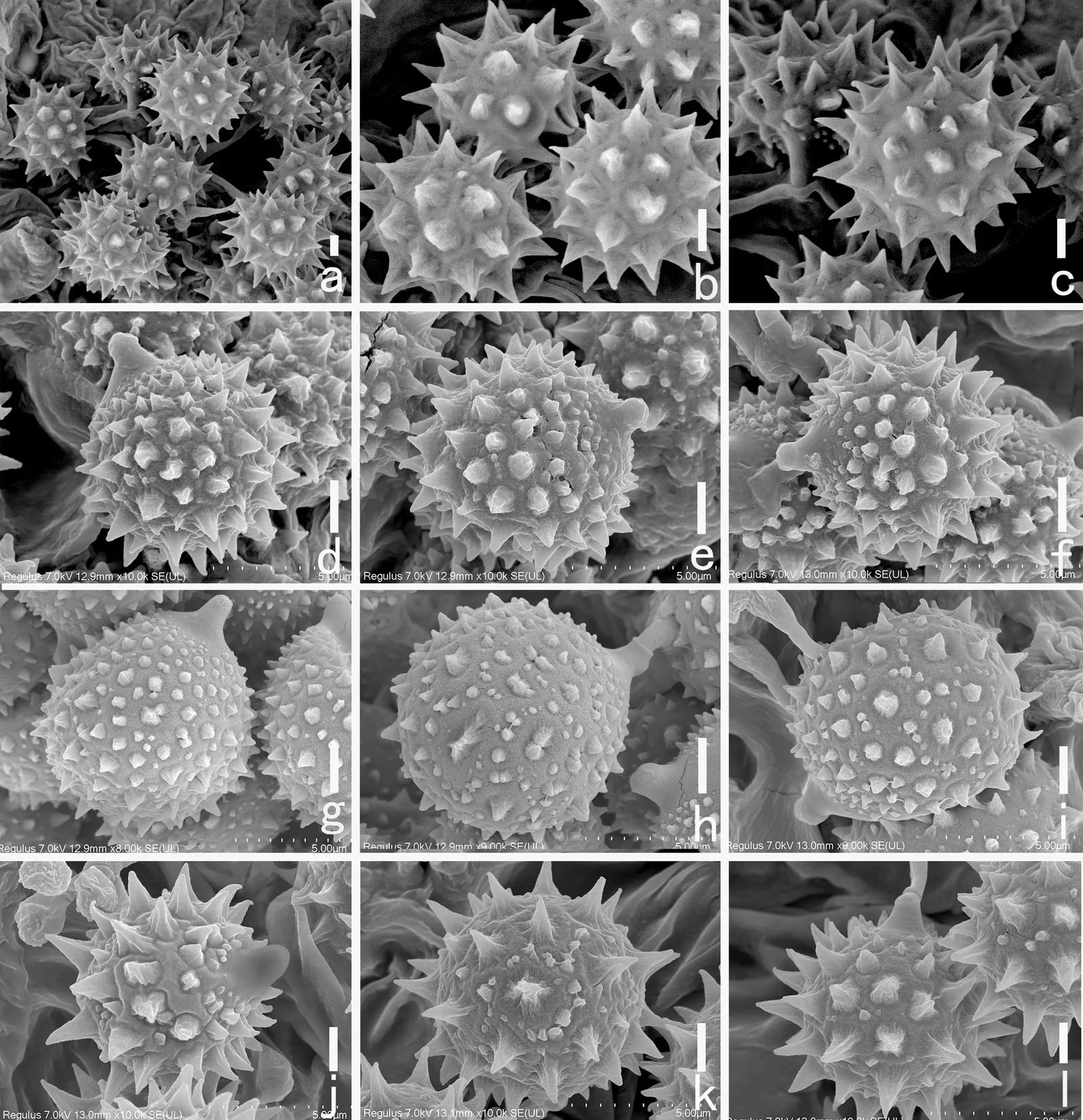
Characteristics of basidiospore ornamentation: **a–c**. *Laccaria violaceonana*; **d–f**. *Laccaria daliensis*; **g–i**. *Laccaria pumila*; **j–l**. *Laccaria acanthospora*. Scale bars: 2 μm.

Edible ectomycorrhizal mushrooms are a group of fungi that form symbiotic relationships with tree roots and produce macroscopic fruiting bodies large enough for manual harvesting, and they have a long tradition of consumption in many regions worldwide (Zambonelli and Bonito 2012). These fungi are found not only in forests but also in grassland ecosystems (Massicotte et al. 1998; Gao and Yang 2010, 2016). Unlike commonly cultivated saprotrophic mushrooms (e.g., *Lentinula edodes*, *Auricularia* spp., *Pleurotus* spp.), their growth and reproduction strictly depend on living host plants (Wei et al. 2021). As an important source of food and medicine, edible ectomycorrhizal mushrooms comprise nearly 1,000 known species worldwide (Hall et al. 2011b, 2016). Among them, the genus *Laccaria* is a typical ectomycorrhizal group, and all currently known species are considered edible. Nutritional analyses indicate that species including *L. laccata* are characterized by high protein and fiber contents and low fat content and are rich in minerals, vitamins, and 17 amino acids (Luo et al. 2018). The Checklist of Edible Fungi in China lists 11 *Laccaria* species (Dai et al. 2010), four of which have been documented to possess antitumor activity (Dai et al. 2008). In Asia, some larger species such as *L. yunnanensis* and *L. moshuijun* are highly valued for their excellent culinary qualities (Tang et al. 2025). *Laccaria daliensis*, *L. violaceonana*, *L. pumila*, and *L. acanthospora* investigated in this study are also edible fungi, further enriching the edible fungal resources of this genus and providing new germplasm materials for their development and utilization.

## Supplementary Material

XML Treatment for Laccaria daliensis

XML Treatment for Laccaria violaceonana

XML Treatment for Laccaria pumila

XML Treatment for Laccaria acanthospora

## Additional information

### Artificial Intelligence (AI) use

The authors accept full responsibility for the content of the manuscript, including the disclosure of any use of AI.

No AI tools were used in the preparation of this manuscript.

### Author ORCIDs

G. Zhao https://orcid.org/0009-0007-9165-6388

H.-N. Suo https://orcid.org/0009-0000-8516-7309

K.-Y. Niu https://orcid.org/0009-0000-8479-890X

Z.-L. Luo https://orcid.org/0000-0001-7307-4885

H.-B. Song https://orcid.org/0000-0002-7440-5444

S.-M. Tang https://orcid.org/0000-0003-0665-6900

### Data availability

All of the data that support the findings of this study are available in the main text or Supplementary Information.
